# *In silico* exploration of potent flavonoids for dengue therapeutics

**DOI:** 10.1371/journal.pone.0301747

**Published:** 2024-12-12

**Authors:** Anuraj Phunyal, Achyut Adhikari, Jhashanath Adhikari Subin

**Affiliations:** 1 Central Department of Chemistry, Tribhuvan University, Kirtipur, Kathmandu, Nepal; 2 Bioinformatics and Cheminformatics Division, Scientific Research and Training Nepal Private Limited, Kaushaltar, Bhaktapur, Nepal; Government College of Engineering, Keonjhar, INDIA

## Abstract

Dengue poses a persistent and widespread threat with no effective antiviral drug available till now. Several inhibitors have been developed by targeting the viral non-structural proteins including methyl transferase (NS5) of the dengue virus with possible therapeutic values. In this work, virtual screening, molecular docking, molecular dynamics simulations (200 ns), and assessments of free energy changes have been carried out to identify potential candidates from a database of flavonoids (*ca*. 2000) that may have good curative potential from the disease. The binding affinity of flavonoids, namely Genistein-7-glucoside (FLD1), 6’–O-Acetylgenistin (FLD2), 5,6-dihydroxy-2-(4-hydroxyphenyl)-7-[3,4,5-trihydroxy-6-(hydroxymethyl)oxane-2-yl]oxychromen-4-one (FLD3), Glucoliquiritigenin (FLD4), and Chrysin-7-O-glucoronide (FLD5) showed the binding affinities of −10.2, −10.2, −10.1, −10.1, −9.9 kcal/mol, respectively, and possessed better values than that of the native ligand (−7.6 kcal/mol) and diclofenac sodium (−7.3 kcal/mol). Drug-likeness of the top five flavonoids were acceptable and no end-point toxicity was hinted by ADMET predictions. The stability of the protein-ligand complexes was accessed from 200 ns molecular dynamics simulations in terms of various geometrical parameters; RMSD, RMSF of residues, Rg, SASA, H-bond, and RPDF. The binding free energy changes of these adducts were calculated by the MM/PBSA solvation model with negative values (from −38.01±7.53 to −17.75±11.03 kcal/mol) indicating the sustained spontaneity of the forward reaction and favorability of the product formation. The geometrical and thermodynamic parameters inferred that the flavonoids could bind at the orthosteric site of the target protein of DENV-2 and could inhibit its functioning, possibly, resulting in the prevention of the disease. Overall, this study highlights the anti-DENV activity of five flavonoids, positioning them as promising candidates for further development as antiviral agents against dengue infection.

## 1. Introduction

The dengue virus (DENV) belongs to the Flaviviridae family and is estimated to impact *ca*. 400 million individuals worldwide annually, primarily through the bite of infectious mosquitoes [1, 2]. The economic burden of dengue is detrimental, costing USD 8.9 billion yearly [3]. Therefore, prevention and cure of this arboviral disease among others is of dire importance. DENV causes dengue fever, dengue hemorrhagic fever (DHF), dengue shock syndrome (DSS), and also damages various organs [4, 5]. Dengue fever exhibits signs in individuals, including a rise in body temperature reaching 40°C, discomfort in muscles and joints, intense headaches, reddening of the face, appearance of skin rashes, and severe flu-like indications [6]. During DHF, a rapid increase in body temperature is observed, and when entering the critical phase, a notable decline in platelets (below 100,000 cells/μL), white blood cells (WBC), and neutrophil levels are diagnosed [7]. The plasma escape during fever in individuals infected with dengue is a critical condition that can result in DSS [8]. Currently, no specific antiviral medications for treating dengue infection have been developed or approved and it poses a tough challenge in the field of prophylactics design. It can be considered a research gap that mandates prompt filling. Sanofi-Pasteur’s tetravalent dengue vaccine (CYD-TDV, Dengvaxia) has been approved for clinical use in several countries [9]. It has raised concerns regarding its overall efficacy in the general population [10]. Therefore, proper, effective, and safe therapeutics for the treatment and cure of dengue are still lacking.

**Fig 1:**
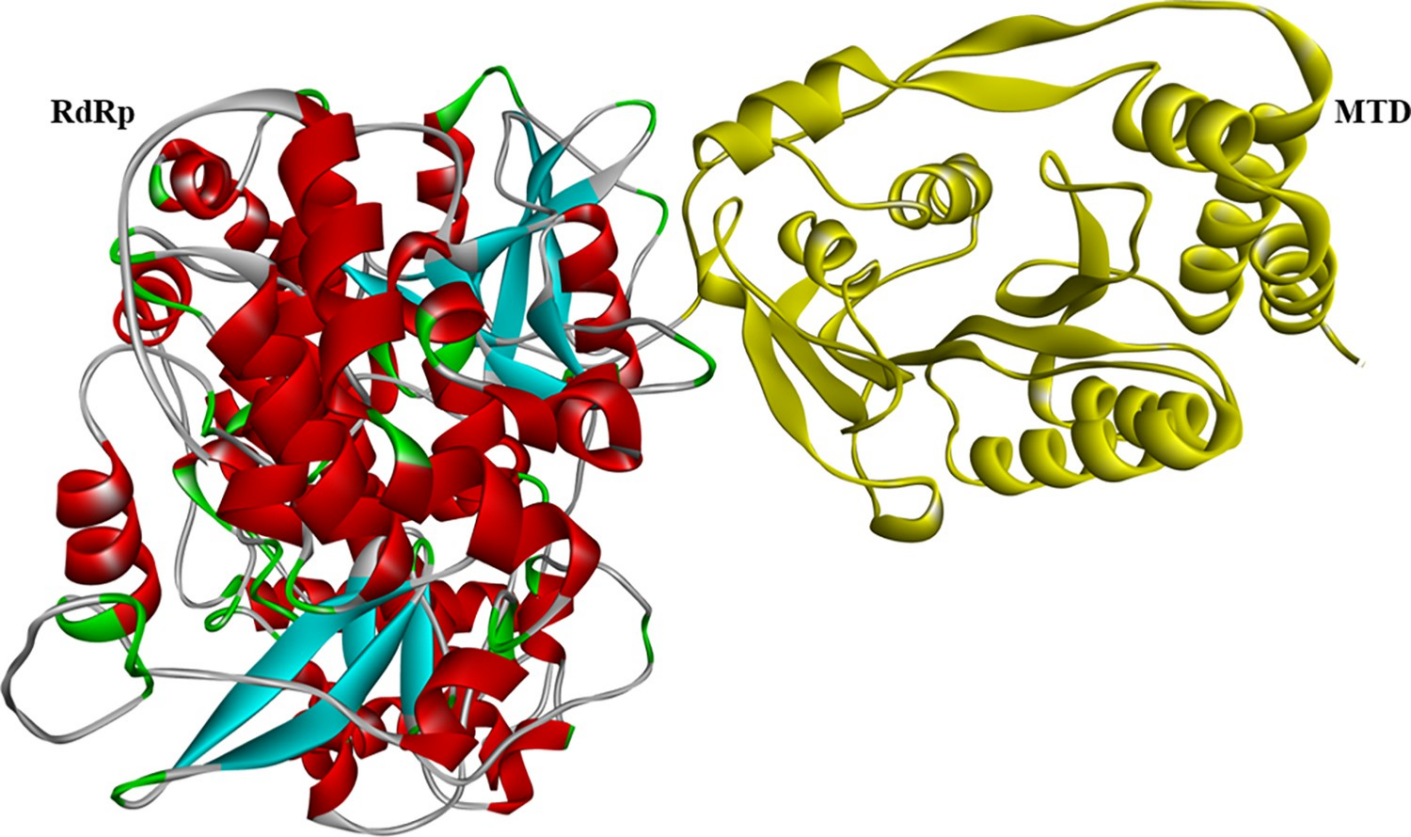
Cartoon representation of the two domains of NS5 protein of dengue virus chosen as a target structure.

DENV has four serotypes (DENV-1, DENV-2, DENV-3, and DENV-4). Among them DENV-2 is also called severe dengue and is commonly reported in Asia and Latin America [11]. The single-stranded positive-sense RNA genome is its genetic material, *ca*. 11 kb in size [12]. Genomic RNA is translated to a polyprotein which produces three structural proteins, capsid (C), pre-membrane (prM), and envelope (E). These are responsible for forming different parts of the virion structure. Additionally, it encodes seven non-structural proteins (NS1, NS2A/B, NS3, NS4A/B, and NS5) that play a pivotal role in viral RNA replication [13]. This article focuses on NS5 protein of DENV-2 which is considered a good and druggable therapeutic target [14]. It is the largest protein, consisting of *ca*. 900 amino acid residues and has two distinct domains: a methyl transferase domain (MTD) located at the N-terminal and an RNA-dependent RNA polymerase domain (RdRp) at the C-terminal (Fig 1).

The MTD plays a crucial role in preventing viral mRNA degradation from 5’-exoribonucleases of the host. It accomplishes this by adding a methyl group to the mRNA, which protects it from being recognized and degraded by host cellular enzymes. The eukaryotic translation initiation factor can recognize the mRNA required for the continuation of its life cycle [15]. At GTP (guanosine triphosphate) binding site, the methylation occurs at guanosine N-7 position, forming N-7- methylguanosine. At another catalytic site called S-adenosylmethionine (SAM) binding site in MTD, methylation takes place at the ribose, also leading to the formation of 2’ -O- methyl-adenosine. SAM (a native ligand) is utilized as the source of methyl groups for this process [16]. It is a secondary metabolite present in virtually all bodily tissues, fluids, and plays a pivotal role in numerous vital functions, including immune system regulation. MTD is a good target and competitive inhibitors of SAM that do not provide methyl groups for protecting mRNA and escaping host immune system are desired for effective therapeutic intervention [17].

Flavonoids, a class of organic molecules, have been reported to possess antiviral activity, making them promising candidates for inhibiting the function of methyl transferase of dengue virus and to cure the disease [18–20]. This work aims to identify NS5 inhibitors from a pool of flavonoids in a low-cost environment. The molecules with the strongest and the most stable binding, with the least or null toxicity, and optimum drug-likeness would be proposed by using different computational methods (molecular docking, molecular dynamics simulation, binding free energy calculations, and ADMET prediction). The results would help in identifying the hit candidates that could be used for further *in vitro* and *in vivo* trials in the drug design and development process.

## 2. Computational experiment

### 2.1. Selection and preparation of ligand database

A dataset of ligands (*ca*. 2000 molecules) was constructed using a similar structure search of the scaffold of a flavonoid and 3D chemical structures were retrieved from the PubChem server in sdf format [21]. The ligands were then prepared for molecular docking by adding polar hydrogen atoms using the Avogadro software v1.1 [22]. After that energy minimization was done with conjugate gradient approach, energy convergence of 10^−8^ units, and with UFF force field for 2000 cycles. The geometry optimization was performed multiple times until no significant structural changes were observed (ΔE = 0). The sdf format of ligand was changed to pdb format by using the PyMOL v2.5.4 program and was further converted to pdbqt format followed by the addition of Gasteiger charges [23]. The bond order and the molecular formula were verified for all the compounds.

### 2.2. Protein structure preparation, homology modeling, and validation

The NS5 protein of DENV, with the PDB ID of 5ZQK (resolution of 2.30Å, X-ray diffraction, expression organism: *E*. *coli*) (https://doi.org/10.2210/pdb5ZQK/pdb) was chosen as the target [24]. The crystal structure was obtained as a pdb file from the Protein Data Bank server (https://www.rcsb.org/). Chain A was chosen and water molecules along with ions and other cofactors were removed. It was followed by the addition of polar hydrogen atoms and Kollman’s charge using AutoDockTools v1.5.7 [25]. It was saved in pdbqt format necessary for molecular docking calculations. Since the protein structure was missing some amino acid residues, homology modeling was performed by using the SWISS-MODEL server [26]. The FASTA sequence was downloaded and the BLAST database search method was employed. A template (5zqk.1.A) was generated and structural descriptors like Global Model Quality Estimation (GMQE) along with Quaternary Structure Quality Estimation (QSQE) were obtained [27, 28]. From the selected template, a 3D protein model was generated. It was converted to pdbqt format and subsequently utilized for molecular docking calculations. The SAVES v6.0 web server (https://saves.mbi.ucla.edu/) with three modules namely, ERRAT, VERIFY3D, and PROCHECK (Ramachandran plot) were used to validate the quality of the protein model [29].

### 2.3. Pharmacodynamics and pharmacokinetics

Absorption, distribution, metabolism, excretion (pharmacokinetics), and toxicity (pharmacodynamics) parameters were calculated from the ADMETlab 2.0, ProTox-Ⅱ, and SwissADME servers, of all the flavonoids and reference drugs (diclofenac sodium) [30–33].

### 2.4. Molecular docking

Molecular docking calculation was performed using the AutoDock Vina v1.1 software, as well as the DockThor and the SwissDock servers for verification of the results [34–36]. In case of first program, AutoDockTools v1.5.7 was used for generating the grid maps of protein and for the visualization. A standard grid box size of 40 Å × 40 Å × 40 Å, the grid center at (57.568, 3.995, and 47.337), an energy range of 4, spacing of 0.375 Å, and the number of modes of 20 with an exhaustiveness of 32 were used. The CASTp server was used to verify the location of the orthosteric site [37]. The top five protein-ligand complexes in terms of binding affinities were saved in pdb format and used for molecular dynamics simulations. Visualization of the binding interaction between protein and ligand was done through Biovia Discovery Studio 2021 software [38].

### 2.5. Molecular dynamics simulation (MDS)

The protein-ligand adduct was simulated by using the GROMACS program, employing the CHARMM27 force field for both the ligand and the receptor [39, 40]. The ligand force field was obtained from the SwissParam server as.zip format [41]. To solvate the system, TIP3P water model was used in a triclinic box (a = 11.504 nm, b = 10.185 nm, c = 11.165 nm, volume = 1308.18 nm^3^) with a spacing of 12 Å at the sides chosen to minimize the spurious interactions between the periodic images [42]. The system was neutralized with counter ions and an isotonic solution of NaCl (0.15 M) was added. The equilibration was processed in four steps, each of 200 ps, at a physiological temperature of 310 K. The first two steps involved NVT ensemble, while the last two steps involved NPT ensemble. During equilibration, temperature coupling (modified Berendsen thermostat), and pressure coupling (Berendsen) were applied, with PME (particle mesh Ewald) used for long-range Coulomb interactions [43, 44]. A production run was conducted for a duration of 200 ns without imposing any restraints with a step size of 2 fs. Geometrical parameters such as RMSD (Root Mean Square Deviation), RMSF (Root Mean Square Fluctuation), Rg (Radius of Gyration), SASA (Solvent Accessible Surface Area), hydrogen bond count, and RPDF (Radial Pair Distribution Function) were extracted from the MDS trajectory using the inbuilt modules of the GROMACS program.

### 2.6. Binding free energy changes by MM/ PBSA method

Molecular mechanics Poisson-Boltzmann surface area (MM/PBSA) method was used in calculating the changes in binding free energies of the protein-ligand complex according to the following equations [45]

$$\Delta G_{BFE}=\Delta G_{complex}-(\Delta G_{protein}+\Delta G_{ligand})$$ (1)

$$\Delta G_{BFE}=\Delta G_{gas}+\Delta G_{solv}$$ (2)

ΔG_gas_ is the sum of electrostatic (ΔE_EL_), and van der Waals” energies (ΔE_VDW_), and the solvation-free energy (ΔG_solv_) is the sum of polar (ΔE_PB_), and nonpolar (ΔE_NPOLAR_) parts [46]

$$\Delta G_{BFE}=\Delta G_{VDW}+\Delta G_{EL}+\Delta G_{PB}+\Delta G_{NPOLAR}$$ (3)

Based on the sign of free energy changes, the forward reaction’s spontaneity and viability were assessed. Here, 200 frames corresponding to 20 ns of the equilibrated part of the MDS trajectory were adopted.

## 3. Results and discussion

### 3.1. Validation of 3D structure of protein and analysis

Before molecular docking, the model of protein was validated for its structural integrity and robustness from server-based calculations. The overall quality factor from the ERRAT module was found to be 94.22, and from the VERIFY module, the structure of the protein was verified with the parameter of 87.02% (more than 80.00% is considered good). From the PROCHECK module, the Ramachandran plot was obtained and it showed over 90% of the residues in the most favored region (S1 Fig). GMQE and QSQE values were determined to be 0.84 and 0.82 respectively, both exceeding the threshold of 0.7. The active site residues were identified as SER79, GLY109, TRP110, LYS128, ASP154, and VAL155. The results of BLAST obtained from the FASTA sequence of protein have provided additional details of the structure (S2 Fig). The results indicated the structure of the protein to be of high quality and was suitable for computational modeling.

### 3.2. Molecular docking protocol validation

Molecular docking protocol validation was done by docking the native ligand at the active site resulting in heavy-atom RMSD of less than 3 Å relative to the pose in the crystal structure (S3 Fig) [47]. This justified the parameters taken, the algorithm used in obtaining the binding affinities, and the calculated ligand poses.

### 3.3. ADMET analysis

Conducting an ADMET study can help reduce the likelihood of failure or later recall of the drug candidates. The drug-likeness, ADME characteristics, and toxicity profile of the potential candidates were evaluated by a server-based approach. Out of *ca*. 2000 flavonoids taken, only 34 successfully passed the criteria (virtually screened). Many candidates (class 5 toxicity, LD_50_ >2500 mg/kg) showed a lack of hepatotoxicity, carcinogenicity, mutagenicity, immunotoxicity, and cytotoxicity (S1 Table). It was found that the candidates exhibited plasma protein binding (PPB) of less than 90%, indicating suitability and a high therapeutic index (S2 Table). Furthermore, it demonstrated acceptable results (ranging from 0 to 0.3) for skin sensitization and minimal eye corrosion/irritation, almost reaching non-sensitizing levels (S3 Table). The central nervous system permeability (logPS) of the hit candidates were determined to be less than −3, indicating impermeability to the CNS (S4 Table). Additionally, the gastrointestinal (GI) effects of many candidates were also found to be acceptable.

The flavonoids would not inhibit CYP2D6, CYP1A2, CYP2C19, CYP2C9, and CYP3A4 enzymes, which play a vital role in metabolizing xenobiotic and minimizing the negative effects of drug interaction [48]. Also, it does not act as a renal Organic Cation Transporter 2 (OCT2) substrate which is therefore unlikely to have a significant impact on renal clearance. Any prediction of toxicity from *in silico* methods mandates verification by different experimental methods. The reference drug, Diclofenac sodium (class 3, LD_50_ 53 mg/kg) demonstrated a remarkably elevated PPB rate of 99.21% and thus possessed a relatively low therapeutic index. It is predicted to induce respiratory toxicity and provoke skin sensitization. The high gastrointestinal absorption exhibited by this drug enables effective system distribution and therapeutic effects but it also possesses hepatotoxic properties. The top five flavonoids showed better drug-likeness and lower toxicity compared to both the native ligand and the reference drug. The results usher towards exploration of further steps in the drug design process with the hit candidates.

### 3.4. Molecular docking analysis

34 molecules that passed the ADMET screening, the native ligand and the reference molecule were subjected to molecular docking calculations against NS5 methyl transferase. The possibility of competitive inhibition was explored. The binding affinities ranging from −10.2 to −7.6 kcal/mol, surpassing those of the native ligand (−7.6 kcal/mol) and reference molecule (−7.3 kcal/mol) were obtained, indicating stronger binding (Table 1). FLD1, FLD2, FLD3, FLD4, and FLD5 could be considered as the top 5 candidates based on the docking score (S4 Fig).

**Table 1:** List of flavonoids with binding affinities from molecular docking calculations.

S.N.	Flavonoids (PubChem CID)	Code	Binding Affinity (kcal/mol)
1	5284639	FLD1	−10.2
2	22288010	FLD2	−10.2
3	14332446	FLD3	−10.1
4	13473703	FLD4	−10.1
5	14135334	FLD5	−9.9
6	5213	FLD6	−9.8
7	13873666	FLD7	−9.8
8	137796321	FLD8	−9.8
9	51666248	FLD9	−9.8
10	74819354	FLD10	−9.7
11	53398699	FLD11	−9.7
12	72747690	FLD12	−9.5
13	12004622	FLD13	−9.5
14	623002	FLD14	−9.5
15	73192461	FLD15	−9.5
16	5358913	FLD16	−9.5
17	5489114	FLD17	−9.5
18	4183640	FLD18	−9.4
19	25202038	FLD19	−9.3
20	51136398	FLD20	−9.3
21	3321055	FLD21	−9.2
22	3733033	FLD22	−9.2
23	5317025	FLD23	−9.2
24	73829903	FLD24	−9.0
25	3483754	FLD25	−8.9
26	74977902	FLD26	−8.9
27	4789	FLD27	−8.8
28	42607667	FLD28	−8.8
29	6169038	FLD29	−8.7
30	131752198	FLD30	−8.7
31	5080434	FLD31	−8.6
32	20979874	FLD32	−8.6
33	102421333	FLD33	−7.9
34	73009474	FLD34	−7.6
35	440426607	Native ligand (SAM)	−7.6
36	5018304	Diclofenac sodium	−7.3

**Fig 2:**
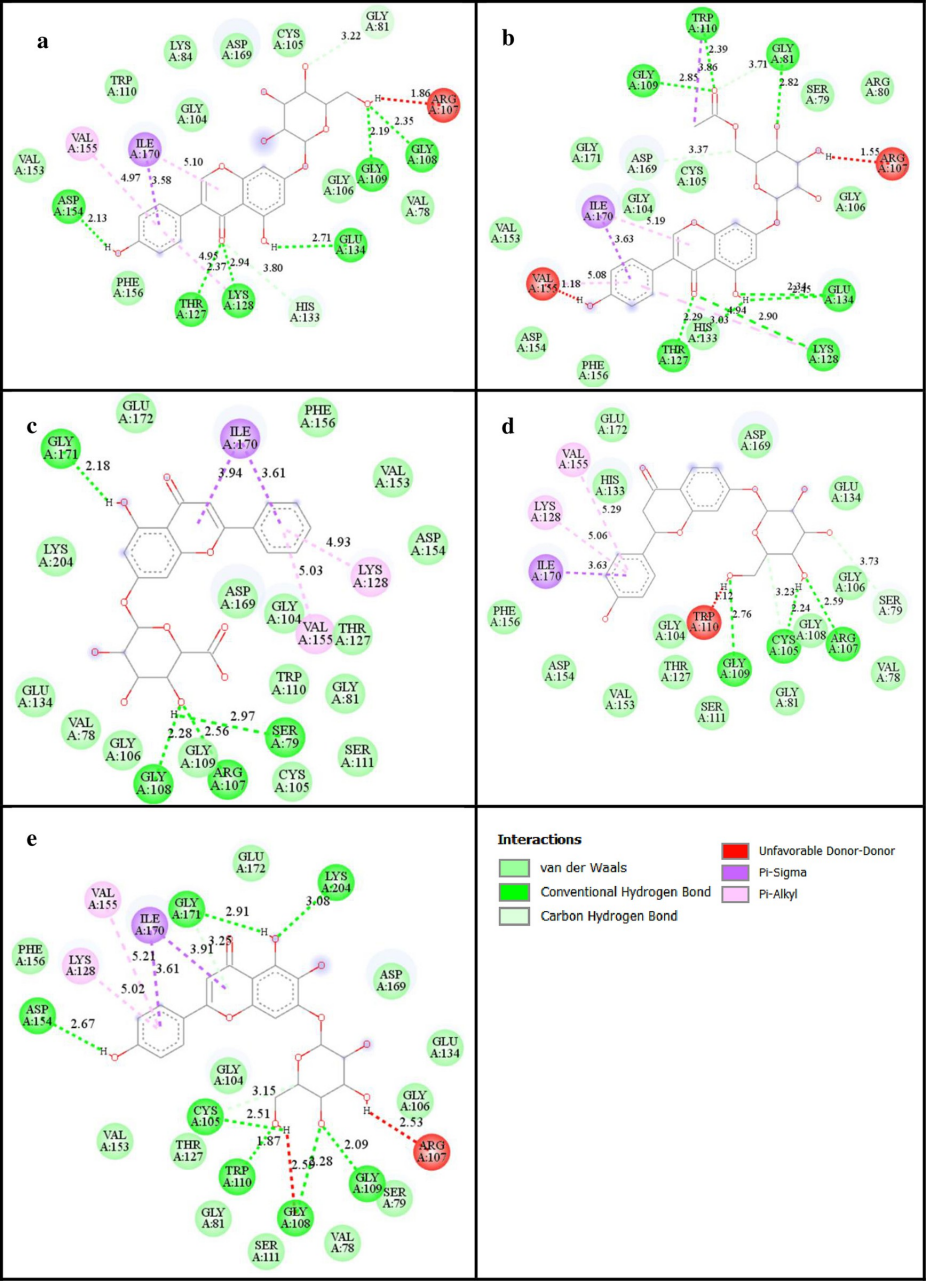
2D projection of interaction of ligands with protein. (a) FLD1 (b) FLD2 (c) FLD3 (d) FLD4, and (e) FLD5.

**Fig 3:**
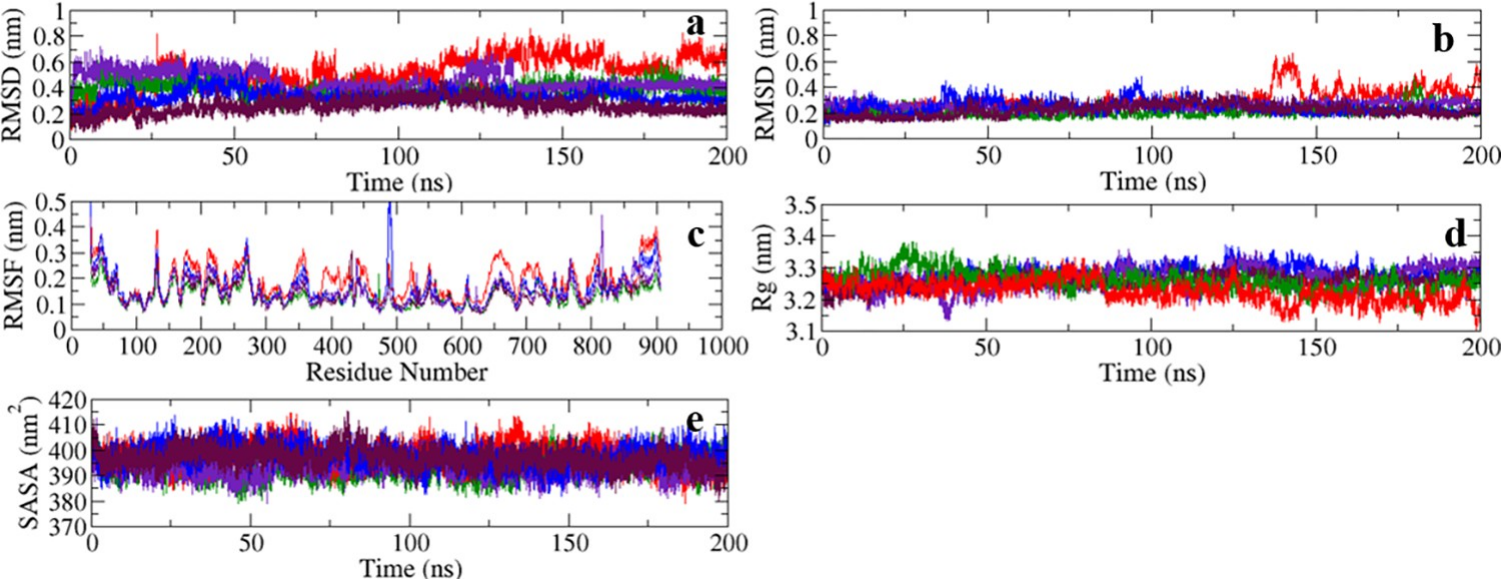
RMSD of (a) ligands relative to protein backbone and (b) protein backbones relative to protein backbone, (c) RMSF curves of α-carbon atoms of protein, (d) Rg curves of protein, and (e) SASA of protein in different complexes obtained from 200 ns MDS trajectories Adducts with flavonoids; Indigo curve = FLD1, red curve = FLD2, blue curve = FLD3, maroon curve = FLD4, green curve = FLD5.

The molecular-level details of bonding interaction with the key amino acid residues by different flavonoids along with distances have been extracted (Table 2). Various types of interactions have been found for five different complexes. Notably, FLD1 and FLD2 showed exemplary binding affinities of −10.2 kcal/mol each, and ranked highest among all the molecules tested. FLD1 formed hydrogen bonds with GLY108 (2.35 Å), **GLY109** (2.19 Å), THR127 (2.37 Å), **LYS128** (2.94 Å), GLU134 (2.71 Å), and **ASP154** (2.13 Å), along with GLY81 (3.22 Å), HIS133 (3.80 Å), ARG107 (1.86 Å), **VAL155** (4.97 Å), and ILE170 (3.80 Å) were additional binding site residues involved in other types of associative interactions. Other 9 residues showed van der Waals” interactions in the complex (Fig 2A). Similarly, FLD2 was engaged in hydrogen bonding with GLY81 (2.82 Å), **GLY109** (2.85 Å), **TRP110** (2.39 Å), THR127 (2.29 Å), **LYS128** (2.90 Å), and GLU134 (2.34 Å). Also other non-covalent interactions with GLY81 (3.71 Å), ARG107 (1.55 Å), **LYS128** (4.94 Å), **VAL155** (1.18 Å), HIS133 (3.03 Å), and ILE170 (3.63 Å) were observed. 11 residues with van der Waals’ interaction could also be seen in the complex (Fig 2B). The presence of multiple hydrogen bonds in these two complexes could be reason for better docking scores. FLD3 and FLD4 exhibited strong binding affinities of −10.1 kcal/mol each with NS5 protein. FLD3, for instance, formed hydrogen bonds with **SER79** (2.97 Å), ARG107 (2.56 Å), GLY108 (2.28 Å), and GLY171 (2.18 Å). Additional interactions were observed with **LYS128** (4.93 Å), **VAL155** (5.03 Å), and ILE170 (3.61 Å) amino acid residues along with 16 van der Waals’ interactions (Fig 2C). The details in the complexes of FLD4 and FLD5 are similar to that of the other three (Fig 3D and 3E). Similar active site residues have been reported in previous studies with the same receptor indicating consistency of the calculations [49, 50]. Despite overall favorable interactions, some unfavorable ones were also present in FLD1, FLD2, FLD4, and FLD5 complexes due to steric hindrance, incompatible geometry, electrostatic repulsion, and solvent effects [51]. Analysis of 3D interactions revealed distinct hydrophobic and hydrophilic regions for the five different adducts (S5 Fig). The brown-colored region indicates hydrophobicity and the blue-colored region denotes hydrophilicity each having nearly compatible type of functional groups of the ligands in their vicinities. The results obtained from two other servers (S5 Table) provide nearly similar inferences as discussed above and henceforth, rationalize the current computational findings. Notably, the top five flavonoids determined from the DockThor server are identical, revealing reproducibility of binding strength from different scoring algorithms. However, the SwissDock server has captured only some top scorers pointing towards the limitations of the use of multiple molecular docking algorithms.

**Table 2:** Different types of interaction and key amino acid residues of protein (PDB ID 5ZQK) in different complexes.

Compounds (PubChem CID)	Interactions	Key amino acid residues	Distance (Å)
**5284639 (FLD1)**	H-bonding	GLY108, **GLY109**, THR127, **LYS128**, GLU134, **ASP154**,	2.35, 2.19, 2.37, 2.94, 2.71, 2.13,
	Alkyl, Pi, ions, Unfavorable	GLY81, HIS133, ARG107, **VAL155**, ILE170	3.22, 3.80, 1.86, 4.97, 3.80
	van der Waals’	VAL78, LYS84, GLY104, CYS105, GLY106, **TRP110**, HIS133, VAL153, PHE156	-
**22288010 (FLD2)**	H-bonding	GLY81, **GLY109**,**TRP110**, THR127, **LYS128**, GLU134	2.82, 2.85, 2.39, 2.29, 2.90, 2.34
	Alkyl, Pi, ions, unfavorable	GLY81, ARG107, **LYS128** **VAL155**, HIS133, ILE170,	3.71, 1.55, 4.94, 1.18, 3.03, 3.63
	van der Waals’	**SER79**, ARG80, GLY104, CYS105, GLY106, HIS133, VAL153, **ASP154**, PHE156, ASP169, GLY171	**-**
**14332446** **(FLD3)**	H-bonding	**SER79**, ARG107, GLY108 GLY171	2.97, 2.56, 2.28, 2.18
	Alkyl, Pi, ions, unfavorable	**LYS128**, **VAL155**, ILE170	4.93, 5.03, 3.61
	van der Waals’	VAL78, GLY81, GLY104, CYS105, GLY106, GLY108, **GLY109**, **TRP110**, SER111 GLU134, VAL153, PHE154, PHE156, ASP169, GLU172 LYS204	-
**13473703** **(FLD4)**	H-bonding	CYS105, ARG107, **GLY109**	2.24, 2.59, 2.76
	Alkyl, Pi, ions, unfavorable	**SER79**, **LYS128**, **VAL155**, **TRP110**, ILE170	3.73, 5.06, 3.29, 1.12, 3.63
	van der Waals’	VAL78, GLY81, GLY104, GLY106, GLY108, SER111, THR127, HIS133, GLU134, **ASP154**, PHE156, ASP169 GLU172	-
**14135334** **(FLD5)**	H-bonding	CYS105, GLY108, **GLY109**, **TRP110**, **ASP154**, GLY171 LYS204	2.51, 2.28, 2.09, 1.87, 2.67, 2.91, 3.08
	Alkyl, Pi, ions, unfavorable	**LYS128**, **VAL155**, ILE170, ARG107, GLY108	5.02, 5.21, 3.91, 2.53, 2.59
	van der Waals’	VAL78, **SER79**, GLY81, GLU106, GLY108, SER111, THR127, GLU134, VAL153, **ASP154**, PHE156	-

### 3.5. Molecular dynamics simulation (MDS)

#### 3.5.1 Root mean square deviation (RMSD)

RMSD profiling of the ligands and that of the backbone were used to analyze the dynamic behavior and stability of the protein-ligand complexes. RMSD of the ligands and those of protein backbones both relative to the protein backbone (least square fitting) of different complexes were extracted from the MDS trajectories (Fig 3A and 3B). The protein backbone exhibited steady RMSD profiles of *ca*. 3 Å for all the cases except for the protein- FLD2 complex. This indicated that the receptor geometry remained stable upon ligand binding and hinted at the druggable nature of the protein. The RMSD profiles of FLD1, FLD3, FLD4, and FLD5 in their complexes showed smooth trajectories with values between 2 Å to 5 Å. After *ca*. 140 ns the system attained equilibrium and maintained it until the end indicating greater and continual stability. The RMSD of the FLD2 ligand relative to the protein backbone varied during the production run and could not be equilibrated. The unstable nature of this complex relative to the other four could be inferred. The four ligands could be considered to be localized in the SAM binding pocket of the protein during MDS and similar outcomes have been reported in the previous study [52]. Different snapshots taken at different moments illustrate how a molecular-level understanding may be attained in terms of geometry and dynamics of different systems (S6 Table).

#### 3.5.2 Snapshots of ligand at the active site of protein during MDS

Snapshots were retrieved during different instantaneous times of MDS to analyze the orientation (rotational motion) and position (translational motion) of the docked ligands (S6 Table). The images at 0, 50, 100, 150, and 200 ns, revealed that most of the ligands remained at the same location but with different orientations with few exceptions. The geometrical behavior at the molecular-level can be analyzed to justify the nature of the RMSD curve of ligand relative to the backbone. In the case of FLD1, the ligand shifted farther apart from the initial position at 50 ns but retained its position at the active site at 100 ns with a change in orientation. It remained at the same location until 200 ns and this behavior is reflected by the nearly smooth RMSD curve after 100 ns. In FLD2, the ligand changed its position and orientation significantly at the active site at 100 and 150 ns, thus causing multiple spikes with irregularities in its RMSD profile. The protein backbone in the protein-FLD2 complex seemed to undergo a significant conformational shift (the alpha-helix at the right part of the image) and was seen as large spikes in the protein backbone RMSD curve. The results indicated that the protein-FLD2 system could not be stabilized at physiological conditions. The images at different times for FLD3, FLD4, and FLD5 complexes denoted minimal delocalization of the ligands with notable rotational motions. Due to the lack of drifting or translational motion from its initial position, the RMSD curves of these ligands were obtained to be nearly smooth in nature. Also, negligible dynamics of the protein backbone were revealed by the snapshots at different instances. The output indicated that the complexes of FLD3, FLD4, and FLD5 were sturdy in nature without undergoing significant disruption. The periodic monitoring of the dynamical behavior of the system provided a clear understanding of the evolution of various molecules that could be correlated to a structural descriptor.

#### 3.5.3 Root mean square fluctuation (RMSF)

The RMSF was calculated from the MDS trajectory for the α-carbon atoms of the protein, and was found to be below 5 Å in most of the complexes indicating the stability of protein geometry (Fig 3C) [53]. The RMSF of *ca*. 4 Å indicated minimal fluctuation in most amino acid residues of the complexes. However, in the protein-FLD3 complex, residues numbered from 475 to 500 exhibited higher fluctuations at *ca*. 5 Å. This was because of the lack of α-helix or β-sheet secondary structures (constrained geometry with reduced degrees of freedom) and the presence of unbound coils/ loops (unrestrained geometry with higher degrees of freedom) [54]. These regions have no significant interactions with the ligand and large RMSF does not indicate instability. The small fluctuations of the active site residue region at 79 to 155 showed the moderate stability of the receptor upon ligand binding and pointed to negligible effect on the destabilization of the complex. Various RMSF curves conferred that the fluctuations of α-carbon atoms would not have a major impact on the ligand’s binding at the active pocket. The adduct’s stability would remain unaltered, which would likely result in an inhibition of regular protein functioning.

#### 3.5.4 Radius of gyration (Rg)

The Rg of protein in various complexes were extracted from the trajectory and it helped to determine the conformational changes of protein structure (Fig 3D). It gives the average distance from the central axis of the macromolecule to all distributed constituents [55]. The Rg ranged from *ca*. 3.17 to 3.30 nm, hinting at the absence of significant expansion or contraction during the production run except in case of FLD2. The Rg curve of protein in the protein-FLD2 complex showed larger changes after 80 ns until the end, inferring to unstable nature of adduct. The results of the other four adducts imply that there was no considerable receptor expansion or shrinkage in response to ligand binding over the simulated period. The hit flavonoids tended to impart structural intactness and possibly aid in the inhibition of the target protein.

#### 3.5.5 Solvent accessible surface area (SASA)

The SASA of protein helps to determine the total wettable area. It was extracted for different structures from the MDS trajectories and was found to lie in the range of 385 to 405 nm^2^ (Fig 3E). The minimal variation seen in the plot due to the lack of notable changes in the surface geometry provided spatial stability to the complexes upon ligand binding. The protein’s hydrophobic portion and its shape remained unchanged which led to the consistency in solvent reachable area [56]. This geometrical descriptor augmented the earlier inference of adduct stability for most of the cases.

#### 3.5.6 Hydrogen bond count

**Fig 4:**
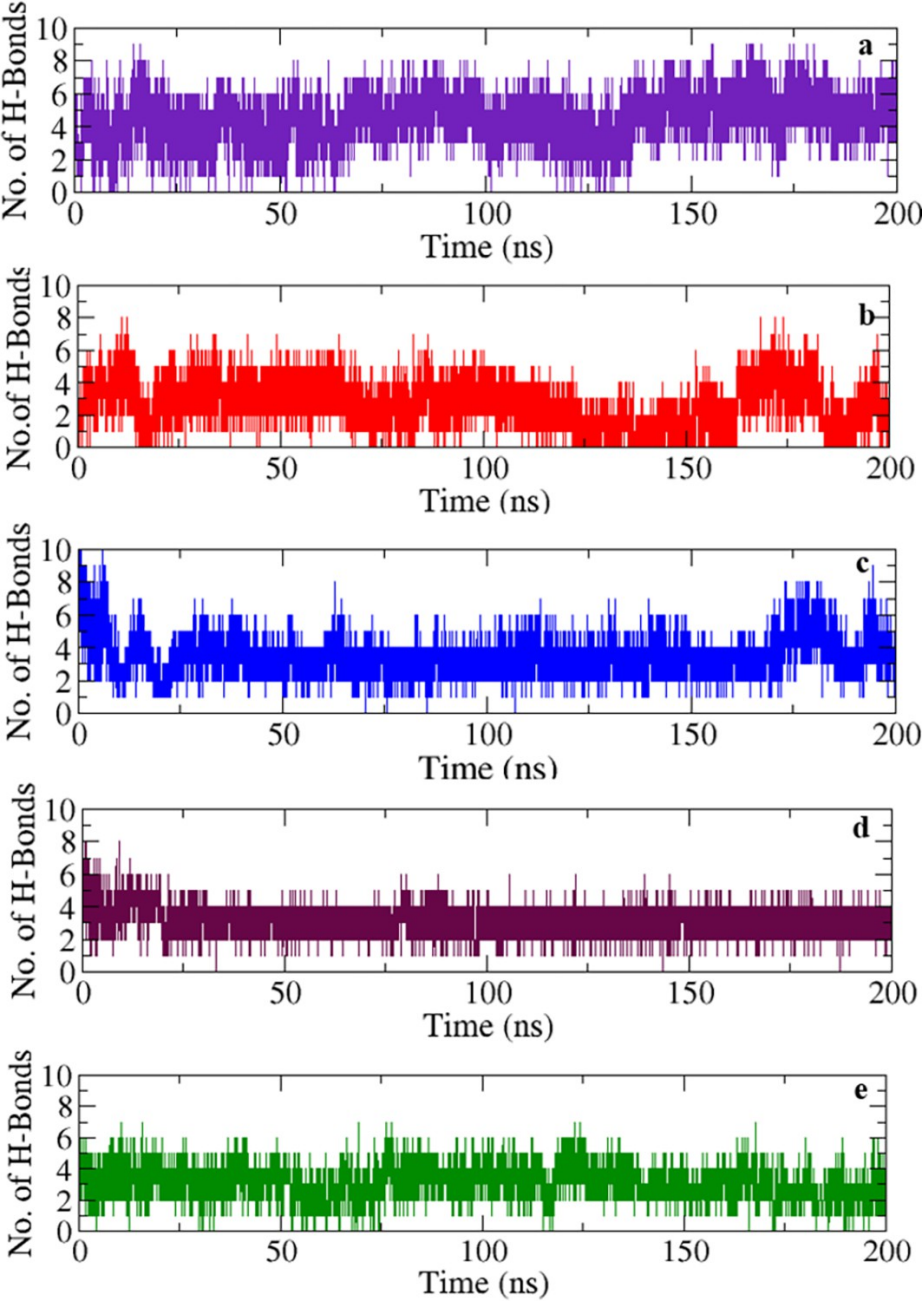
Number of hydrogen bonds between protein and ligands in different complexes monitored during 200 ns production runs. Adducts with flavonoids; Indigo line = FLD1, red line = FLD2, blue line = FLD3, maroon line = FLD4, green line = FLD5.

The variation of hydrogen bond count formed between the ligands and protein during the MDS plays a pivotal role in assessing the stability of complexes [57]. The larger the number of hydrogen bonds (non-covalent interactions), the greater is the stability of the complex. The highest hydrogen bond count of up to 8 several times during the MDS was observed in the protein-FLD1 complex (Fig 4A). Similarly, the protein-FLD2 complex showed a count of up to 6 hydrogen bonds but with notable variation (Fig 4B). The complexes with FLD3, FLD4, and FLD5 displayed 4 or more hydrogen bonds (Fig 4C–4E). In the case of protein with FLD4, a consistent curve in the hydrogen bond count was observed, indicating its relationship with ligand RMSD (smooth RMSD curve, maroon) (Fig 3). The RMSD of FLD1, FLD3, FLD4, and FLD5 ligands remained below *ca*. 4 Å due to restricted translational and rotational motion because of near consistency in hydrogen bond count. However, the RMSD of FLD2 was above 4 Å and may be attributed to variation in hydrogen bond count with time. The results indicated that both the number of hydrogen bond counts and its maintenance along with other controlling factors played a key role in maintaining the stability of the protein-ligand systems.

#### 3.5.7 Radial pair distribution function (RPDF)

**Fig 5:**
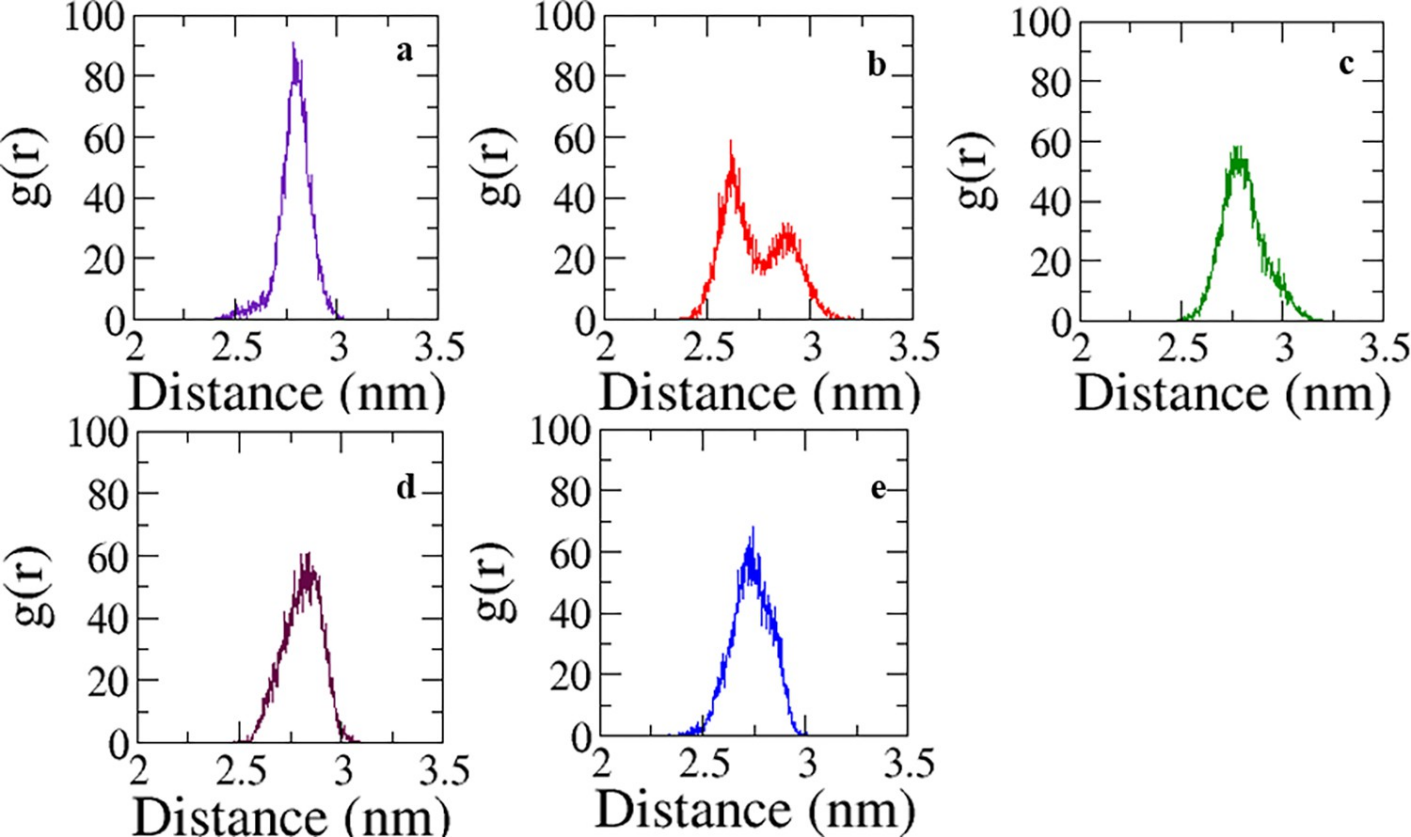
The radial pair distribution function of the ligand and protein in five different complexes extracted from MDS trajectory. (a) FLD1, (b) FLD2, (c) FLD3, (d) FLD4, and (e) FLD5; a sharp single peak indicates the localization of the ligand at a definite site in the protein pointing towards the spatial stability of the adduct.

The modulation of distance between two entities over time is provided by RPDF. The plots for various protein-ligand complexes have been extracted from the MDS trajectories. The occurrence of single sharp peak (maxima at *ca*. 2.75 nm) for FLD1 complexes indicated localization of center of mass of ligand relative to center of mass of the protein (Fig 5A). The presence of two peaks in case of protein-FLD2 complex clearly showed the occupancy at two locations (Fig 5B). Similar results have been reported in the literature [58]. In case of FLD3 (*ca*. 2.76 nm), FLD4 (*ca*. 2.76 nm), and FLD5 (*ca*. 2.74 nm) ligands, a single and a slightly broader peak point towards localization but to a lesser degree (Fig 5C–5E). Therefore, RPDF (fate of ligand with time relative to protein) was able to determine the stability of different adducts through different mathematical formulations and could eventually support the inferences derived earlier by multiple parameters.

### 3.6. Thermodynamic spontaneity and stability

The MM/PBSA calculation utilizing the generalized Born model (GBn) was employed to determine the binding free energy differences (ΔG_BFE_) for the equilibrated portion of the trajectory (20 ns, 200 frames) (Table 3) for different adducts [59].

**Table 3:** Change in binding free energies (kcal/mol) of complexes with different components.

Code	ΔE_VDW_	ΔE_EL_	ΔE_PB_	ΔE_NPOLAR_	ΔG_GAS_	ΔG_SOLV_	ΔG_BFE_
**FLD1**	−36.07±3.31	−37.87±12.24	43.87±7.54	−4.3±0.11	−73.94±10.79	39.57±7.54	−34.38±5.32
**FLD2**	−42.37±2.69	−14.89±16.74	44.07±20.72	−4.56±0.26	−57.26±16.62	39.51v20.52	−17.75±11.03
**FLD3**	−45.67±3.16	−55.73±15.29	67.86±11.88	−4.47±0.10	−101.40±15.38	63.39±11.84	−38.01±7.53
**FLD4**	−44.95±2.63	−40.12±5.35	56.55±5.62	−4.39±0.09	−85.07±5.20	52.16±5.60	−32.91±5.32
**FLD5**	−34.49±4.29	−33.49±14.29	50.29±11.10	−4.77±0.14	−67.99±12.84	45.52±11.06	−22.47±6.12

The negative values of binding free energy changes (ΔGBFE) indicated feasibility of the adduct formation in all the simulated complexes. The lowest binding free energy change (kcal/mol) was observed with the protein-FLD3 complex (−38.01±7.53), followed by the complexes of FLD1 (−34.38±5.32), FLD4 (−32.91±5.32), FLD5 (−22.47±6.12), and FLD2 (−17.75±11.03). The frame–by–frame, ΔG_BFE_ of 20 ns of the equilibrated part of the trajectory is depicted (S6 Fig). The moving average was always negative for most of the complexes implying sustained thermodynamic spontaneity of the reactions in the forward direction. An exception was seen for the protein-FLD2 complex for a very small period. The spontaneous nature of the complex formation reaction indicated that the ligand and the protein preferred to form a complex instead of remaining unattached. Consequently, the interactions between the receptor (PDB ID: 5ZQK), and the flavonoids (FLD1, FLD3, FLD4, and FLD5) were deemed favorable, ensuring the stability of the protein-ligand complexes throughout the production run.

The hit candidates formed spatially and thermodynamically stable protein-ligand complexes as determined from various descriptors extracted from MDS trajectories (cumulative 1 μs). These could notably inhibit or modulate the functioning of methyl transferase by hindering the transfer of methyl group to NS5 protein required for the survival of virus. The preliminary results point towards the requirement of experimental verification for the confirmation of the inferences.

## 4. Conclusions

The potential of flavonoids to inhibit a promising target, NS5 methyl transferase protein of dengue virus (DENV-2), was investigated by different computational methods. Out of 34 flavonoids screened, 33 exhibited higher binding affinity with NS5 protein compared to both the native ligand and a reference drug, diclofenac sodium (employed to lessen the symptoms only). MDS of the top 5 protein-ligand candidates showed good structural stability. The thermodynamical consideration hinted at the sustained spontaneity of the complex formation reaction and the maintenance of the ligand’s pose at the orthosteric site of the receptor. Based on the preliminary results (lowest deviation below 2 Å, smoothest RMSD profile with *ca*. 0.5 Å variation, and comparable free energy changes of −32.91±5.32 kcal/mol), FLD4 could be considered a hit molecule. However, all the top candidates, FLD1, FLD3, FLD4, and FLD5 showed promising potential and are recommended for additional *in vitro* and *in vivo* trials to warrant their inhibition capabilities against dengue protein.

## Supporting information

S1 Fig Structure of protein validation from SAVES 6.0 server. (TIF)

S2 Fig Model-Template alignment of protein. (TIF)

S3 Fig Superimposition of co-crystallized ligand (yellow) and docked ligand (grey-red) obtained from calculations. Heavy-atom RMSD = 2.618 Å.
(TIF)

S4 Fig 2D chemical structures of top five ligands. Based on binding affinity. (TIF)

S5 Fig Docking pose of ligands in the cavity with a hydrophobic surface of protein. (a) FLD1 (b) FLD2 (c) FLD3 (d) FLD4, and (e) FLD5.
(TIF)

S6 Fig Variation of binding free energy changes of various adducts of protein with FLD1, (b) FLD2, (c) FLD3, (d) FLD4, and (e) FLD5. The moving average is shown in red. (TIF)

S1 Table Toxicity of the compounds from ProTox-Ⅱ. (DOCX)

S2 Table ADMET properties of the compounds from ADMETlab 2.0. (DOCX)

S3 Table Toxicity from ADMElab 2.0. (DOCX)

S4 Table ADMET properties from pkCSM server. (DOCX)

S5 Table Binding affinity of compounds from servers. (DOCX)

S6 Table Snapshots of five protein-ligand complexes at different times during MDS. Orientation and location of ligands relative to protein backbone are to be monitored; ligand is shown in ball and stick model and protein in cartoon representation.
(DOCX)

## References

[1] Bhatt S, Gething PW, Brady OJ, Messina JP, Farlow AW, Moyes CL, et al. The global distribution and burden of dengue. Nature. 2013 Apr 25;496(7446):504–7. doi: 10.1038/nature12060 23563266 PMC3651993

[2] Mohapatra RK, Bhattacharjee P, Desai DN, Kandi V, Sarangi AK, Mishra S, et al. Global health concern on the rising dengue and chikungunya cases in the American regions: Countermeasures and preparedness. Health Sci Rep. 2024 Jan;7(1):e1831. doi: 10.1002/hsr2.1831 38274135 PMC10808844

[3] Hung TM, Shepard DS, Bettis AA, Nguyen HA, McBride A, Clapham HE, et al. Productivity costs from a dengue episode in Asia: a systematic literature review. BMC Infect Dis. 2020 Dec;20(1):393. doi: 10.1186/s12879-020-05109-0 32493234 PMC7268537

[4] Behnam MAM, Graf D, Bartenschlager R, Zlotos DP, Klein CD. Discovery of Nanomolar Dengue and West Nile Virus Protease Inhibitors Containing a 4-Benzyloxyphenylglycine Residue. J Med Chem. 2015 Dec 10;58(23):9354–70. doi: 10.1021/acs.jmedchem.5b01441 26562070

[5] Jal S, Chhotaray S, Pattnaik G, Mishra S, Mohapatra RK, Kandi V, et al. Dengue fever coinfection in COVID‐19 era: A public health concern. Health Sci Rep. 2024 May;7(5):e2089. doi: 10.1002/hsr2.2089 38784250 PMC11111600

[6] Luo D, Xu T, Hunke C, Grüber G, Vasudevan SG, Lescar J. Crystal Structure of the NS3 Protease-Helicase from Dengue Virus. J Virol. 2008 Jan;82(1):173–83. doi: 10.1128/JVI.01788-07 17942558 PMC2224403

[7] Potts JA, Rothman AL. Clinical and laboratory features that distinguish dengue from other febrile illnesses in endemic populations. Trop Med Int Health. 2008 Nov;13(11):1328–40. doi: 10.1111/j.1365-3156.2008.02151.x 18803612 PMC2756447

[8] Butthep P, Chunhakan S, Yoksan S, Tangnararatchakit K, Chuansumrit A. Alteration of Cytokines and Chemokines During Febrile Episodes Associated With Endothelial Cell Damage and Plasma Leakage in Dengue Hemorrhagic Fever. Pediatr Infect Dis J. 2012 Dec;31(12):e232. doi: 10.1097/INF.0b013e31826fd456 22926216

[9] Kariyawasam R, Lachman M, Mansuri S, Chakrabarti S, Boggild AK. A dengue vaccine whirlwind update. Ther Adv Infect Dis. 2023 Jan;10:204993612311672. doi: 10.1177/20499361231167274 37114191 PMC10126642

[10] Diaz-Quijano FA, Siqueira De Carvalho D, Raboni SM, Shimakura SE, Maron De Mello A, Vieira Da Costa-Ribeiro MC, et al. Effectiveness of mass dengue vaccination with CYD-TDV (Dengvaxia®) in the state of Paraná, Brazil: integrating case-cohort and case-control designs. Lancet Reg Health—Am. 2024 Jul;35:100777. doi: 10.1016/j.lana.2024.100777 38807985 PMC11131085

[11] Vicente CR, Herbinger KH, Fröschl G, Malta Romano C, De Souza Areias Cabidelle A, Cerutti Junior C. Serotype influences on dengue severity: a cross-sectional study on 485 confirmed dengue cases in Vitória, Brazil. BMC Infect Dis. 2016 Dec;16(1):320. doi: 10.1186/s12879-016-1668-y 27393011 PMC4938938

[12] Islam MdA Hemo MK, Marzan AA Arman MdS, Hasan MN Haque MA, et al. A short communication of 2022 dengue outbreak in Bangladesh: a continuous public health threat. Ann Med Surg. 2023 Jun;85(6):3213–7. doi: 10.1097/MS9.0000000000000623 37363468 PMC10289534

[13] Zhou Z, Khaliq M, Suk JE, Patkar C, Li L, Kuhn RJ, et al. Antiviral compounds discovered by virtual screening of small-molecule libraries against dengue virus E protein. ACS Chem Biol. 2008 Dec 19;3(12):765–75. doi: 10.1021/cb800176t 19053243 PMC2782732

[14] Nawaz A, Ijaz B. Antiviral screening *Azadiricta indica* phytochemicals as dengue NS5 inhibitor: a molecular docking approach. Biol Clin Sci Res J. 2023 Nov 27;2023(1):560. 10.54112/bcsrj.v2023i1.560

[15] Decroly E, Ferron F, Lescar J, Canard B. Conventional and unconventional mechanisms for capping viral mRNA. Nat Rev Microbiol. 2011 Dec 5;10(1):51–65. doi: 10.1038/nrmicro2675 22138959 PMC7097100

[16] Ray D, Shah A, Tilgner M, Guo Y, Zhao Y, Dong H, et al. West Nile Virus 5′-Cap Structure Is Formed by Sequential Guanine N-7 and Ribose 2′-O Methylations by Nonstructural Protein 5. J Virol. 2006 Sep;80(17):8362–70. doi: 10.1128/JVI.00814-06 16912287 PMC1563844

[17] Duan W, Song H, Wang H, Chai Y, Su C, Qi J, et al. The crystal structure of Zika virus NS5 reveals conserved drug targets. EMBO J. 2017 Apr 3;36(7):919–33. doi: 10.15252/embj.201696241 28254839 PMC5376968

[18] Mohapatra PK, Chopdar KS, Dash GC, Mohanty AK, Raval MK. In silico screening and covalent binding of phytochemicals of Ocimum sanctum against SARS-CoV-2 (COVID 19) main protease. J Biomol Struct Dyn. 2023 Jan 22;41(2):435–44. doi: 10.1080/07391102.2021.2007170 34821198

[19] Thames JE, Waters CD, Valle C, Bassetto M, Aouadi W, Martin B, et al. Synthesis and biological evaluation of novel flexible nucleoside analogues that inhibit flavivirus replication in vitro. Bioorg Med Chem. 2020 Nov;28(22):115713. doi: 10.1016/j.bmc.2020.115713 33128910 PMC7457965

[20] Ninfali P, Antonelli A, Magnani M, Scarpa ES. Antiviral Properties of Flavonoids and Delivery Strategies. Nutrients. 2020 Aug 21;12(9):2534. doi: 10.3390/nu12092534 32825564 PMC7551920

[21] Kim S, Chen J, Cheng T, Gindulyte A, He J, He S, et al. PubChem 2023 update. Nucleic Acids Res. 2023 Jan 6; 51(D1):D1373–80. doi: 10.1093/nar/gkac956 36305812 PMC9825602

[22] Hanwell MD, Curtis DE, Lonie DC, Vandermeersch T, Zurek E, Hutchison GR. Avogadro: an advanced semantic chemical editor, visualization, and analysis platform. J Cheminformatics. 2012 Dec;4(1):17. doi: 10.1186/1758-2946-4-17 22889332 PMC3542060

[23] Schro¨dinger LLC, Warren DL. PyMOL. Version 2.4.0.

[24] El Sahili A, Soh TS, Schiltz J, Gharbi-Ayachi A, Seh CC, Shi PY, et al. NS5 from Dengue Virus Serotype 2 Can Adopt a Conformation Analogous to That of Its Zika Virus and Japanese Encephalitis Virus Homologues. Heise MT, editor. J Virol. 2019 Dec 12;94(1):e01294–19. doi: 10.1128/JVI.01294-19 31597763 PMC6912093

[25] Morris GM, Huey R, Lindstrom W, Sanner MF, Belew RK, Goodsell DS, et al. AutoDock4 and AutoDockTools4: Automated docking with selective receptor flexibility. J Comput Chem. 2009 Dec;30(16):2785–91. doi: 10.1002/jcc.21256 19399780 PMC2760638

[26] Waterhouse A, Bertoni M, Bienert S, Studer G, Tauriello G, Gumienny R, et al. SWISS-MODEL: homology modelling of protein structures and complexes. Nucleic Acids Res. 2018;46(W1):W296–303. doi: 10.1093/nar/gky427 29788355 PMC6030848

[27] Biasini M, Bienert S, Waterhouse A, Arnold K, Studer G, Schmidt T, et al. SWISS-MODEL: modelling protein tertiary and quaternary structure using evolutionary information. Nucleic Acids Res. 2014 Jul 1;42(W1):W252–8. doi: 10.1093/nar/gku340 24782522 PMC4086089

[28] Biasini M, Schmidt T, Bienert S, Mariani V, Studer G, Haas J, et al. *OpenStructure*: an integrated software framework for computational structural biology. Acta Crystallogr D Biol Crystallogr. 2013 May 1;69(5):701–9. doi: 10.1107/S0907444913007051 23633579 PMC3640466

[29] Colovos C, Yeates TO. Verification of protein structures: Patterns of nonbonded atomic interactions. Protein Sci. 1993 Sep;2(9):1511–9. doi: 10.1002/pro.5560020916 8401235 PMC2142462

[30] Banerjee P, Eckert AO, Schrey AK, Preissner R. ProTox-II: a webserver for the prediction of toxicity of chemicals. Nucleic Acids Res. 2018 Jul 2;46(W1):W257–63. doi: 10.1093/nar/gky318 29718510 PMC6031011

[31] Daina A, Michielin O, Zoete V. SwissADME: a free web tool to evaluate pharmacokinetics, drug-likeness and medicinal chemistry friendliness of small molecules. Sci Rep. 2017 Mar 3;7(1):42717. doi: 10.1038/srep42717 28256516 PMC5335600

[32] Xiong G, Wu Z, Yi J, Fu L, Yang Z, Hsieh C, et al. ADMETlab 2.0: an integrated online platform for accurate and comprehensive predictions of ADMET properties. Nucleic Acids Res. 2021 Jul 2;49(W1):W5–14. doi: 10.1093/nar/gkab255 33893803 PMC8262709

[33] Ullah A, Atia-tul-Wahab, Gong P, Khan AM, Choudhary MI. Identification of new inhibitors of NS5 from dengue virus using saturation transfer difference (STD-NMR) and molecular docking studies. RSC Adv. 2023;13(1):355–69. doi: 10.1039/d2ra04836a 36605638 PMC9768849

[34] Trott O, Olson AJ. AutoDock Vina: improving the speed and accuracy of docking with a new scoring function, efficient optimization and multithreading. J Comput Chem. 2010 Jan 30;31(2):455–61. doi: 10.1002/jcc.21334 19499576 PMC3041641

[35] Guedes IA, Pereira Da Silva MM, Galheigo M, Krempser E, De Magalhães CS, Correa Barbosa HJ, et al. DockThor-VS: A Free Platform for Receptor-Ligand Virtual Screening. J Mol Biol. 2024 Mar;168548. doi: 10.1016/j.jmb.2024.168548 39237203

[36] Bugnon M, Röhrig UF, Goullieux M, Perez MAS, Daina A, Michielin O, et al. SwissDock 2024: major enhancements for small-molecule docking with Attracting Cavities and AutoDock Vina. Nucleic Acids Res. 2024 Jul 5;52(W1):W324–32. doi: 10.1093/nar/gkae300 38686803 PMC11223881

[37] Tian W, Chen C, Lei X, Zhao J, Liang J. CASTp 3.0: computed atlas of surface topography of proteins. Nucleic Acids Res. 2018 Jul 2;46(W1):W363–7. doi: 10.1093/nar/gky473 29860391 PMC6031066

[38] BIOVIA DS. Discovery Studio Visualizer; v21. 1.0. 20298 Dassault Systèmes: San Diego. CA, USA. 2021.

[39] Abraham MJ, Murtola T, Schulz R, Páll S, Smith JC, Hess B, et al. GROMACS: High performance molecular simulations through multi-level parallelism from laptops to supercomputers. SoftwareX. 2015 Sep;1–2:19–25. 10.1016/j.softx.2015.06.001

[40] Bjelkmar P, Larsson P, Cuendet MA, Hess B, Lindahl E. Implementation of the CHARMM Force Field in GROMACS: Analysis of Protein Stability Effects from Correction Maps, Virtual Interaction Sites, and Water Models. J Chem Theory Comput. 2010 Feb 9;6(2):459–66. doi: 10.1021/ct900549r 26617301

[41] Zoete V, Cuendet MA, Grosdidier A, Michielin O. SwissParam: A fast force field generation tool for small organic molecules. J Comput Chem. 2011;32(11):2359–68. doi: 10.1002/jcc.21816 21541964

[42] Jorgensen WL, Chandrasekhar J, Madura JD, Impey RW, Klein ML. Comparison of simple potential functions for simulating liquid water. J Chem Phys. 1983;79(2):926–35.

[43] Toukmaji A, Sagui C, Board J, Darden T. Efficient particle-mesh Ewald based approach to fixed and induced dipolar interactions. J Chem Phys. 2000 Dec 22;113(24):10913–27. doi: 10.1002/jcc.21816 21541964

[44] Basconi JE, Shirts MR. Effects of Temperature Control Algorithms on Transport Properties and Kinetics in Molecular Dynamics Simulations. J Chem Theory Comput. 2013 Jul 9;9(7):2887–99. doi: 10.1021/ct400109a 26583973

[45] Valdés-Tresanco MS, Valdés-Tresanco ME, Valiente PA, Moreno E. gmx_MMPBSA: A New Tool to Perform End-State Free Energy Calculations with GROMACS. J Chem Theory Comput. 2021 Oct 12;17(10):6281–91. doi: 10.1021/acs.jctc.1c00645 34586825

[46] Kumari R, Kumar R, Lynn A. g_mmpbsa—A GROMACS Tool for High-Throughput MM-PBSA Calculations. J Chem Inf Model. 2014 Jul 28;54(7):1951–62. doi: 10.1021/ci500020m 24850022

[47] Ramírez D, Caballero J. Is It Reliable to Take the Molecular Docking Top Scoring Position as the Best Solution without Considering Available Structural Data? Molecules. 2018 Apr 28;23(5):1038. doi: 10.3390/molecules23051038 29710787 PMC6102569

[48] Basnet S, Ghimire MP, Lamichhane TR, Adhikari R, Adhikari A. Identification of potential human pancreatic α-amylase inhibitors from natural products by molecular docking, MM/GBSA calculations, MD simulations, and ADMET analysis. PloS One. 2023;18(3):e0275765. doi: 10.1371/journal.pone.0275765 36928801 PMC10019617

[49] Tambunan USF, Zahroh H, Utomo BB, Parikesit AA. Screening of commercial cyclic peptide as inhibitor NS5 methyltransferase of Dengue virus through Molecular Docking and Molecular Dynamics Simulation. Bioinformation. 2014 Jan 29;10(1):23–7. doi: 10.6026/97320630010023 24516322 PMC3916815

[50] Alwabli AS, Qadri I. Identification of Possible Inhibitor Molecule against NS5 MTase and RdRp Protein of Dengue Virus in Saudi Arabia. Indian J Pharm Educ Res. 2021;55(4):1028–36.

[51] Regan CK, Craig SL, Brauman JI. Steric effects and solvent effects in ionic reactions. Science. 2002 Mar 22;295(5563):2245–7. doi: 10.1126/science.1068849 11910104

[52] Jarerattanachat V, Boonarkart C, Hannongbua S, Auewarakul P, Ardkhean R. In silico and in vitro studies of potential inhibitors against Dengue viral protein NS5 Methyl Transferase from Ginseng and Notoginseng. J Tradit Complement Med. 2023 Jan 1;13(1):1–10. doi: 10.1016/j.jtcme.2022.12.002 36685072 PMC9845645

[53] Aljarba NH, Hasnain MS, Bin-Meferij MM, Alkahtani S. An in-silico investigation of potential natural polyphenols for the targeting of COVID main protease inhibitor. J King Saud Univ—Sci. 2022 Oct;34(7):102214. doi: 10.1016/j.jksus.2022.102214 35811756 PMC9250415

[54] Sharma BP, Subin JA, Marasini BP, Adhikari R, Pandey SK, Sharma ML. Triazole based Schiff bases and their oxovanadium (IV) complexes: Synthesis, characterization, antibacterial assay, and computational assessments. Heliyon 2023 Dec 10];9(4). doi: 10.1016/j.heliyon.2023.e15239 37089299 PMC10119765

[55] Liu P, Lu J, Yu H, Ren N, Lockwood FE, Wang QJ. Lubricant shear thinning behavior correlated with variation of radius of gyration via molecular dynamics simulations. J Chem Phys. 2017 Aug 30;147(8):084904. doi: 10.1063/1.4986552 28863549

[56] Zhang D, Lazim R. Application of conventional molecular dynamics simulation in evaluating the stability of apomyoglobin in urea solution. Sci Rep. 2017 Mar 16;7(1):44651. doi: 10.1038/srep44651 28300210 PMC5353640

[57] Chikalov I, Yao P, Moshkov M, Latombe JC. Learning probabilistic models of hydrogen bond stability from molecular dynamics simulation trajectories. BMC Bioinformatics. 2011 Dec;12(S1):S34. doi: 10.1186/1471-2105-12-S1-S34 21342565 PMC3044290

[58] Subin JA, Shrestha RL. Computational Assessment of the Phytochemicals of Panax ginseng CA Meyer Against Dopamine Receptor D1 for Early Huntington’s Disease Prophylactics. Cell Biochemistry and Biophys. 2024 Jul 24:1–1. doi: 10.1007/s12013-024-01426-2 39046621

[59] Mongan J, Simmerling C, McCammon JA, Case DA, Onufriev A. Generalized Born Model with a Simple, Robust Molecular Volume Correction. J Chem Theory Comput. 2007 Jan 1;3(1):156–69. doi: 10.1021/ct600085e 21072141 PMC2975579

