# Supplementary figures and images for: In silico exploration of potent flavonoids for dengue therapeutics
Source: PLoS One. 2024 Dec 12;19(12):e0301747. doi: 10.1371/journal.pone.0301747 (PMC11637399; doi:10.1371/journal.pone.0301747)

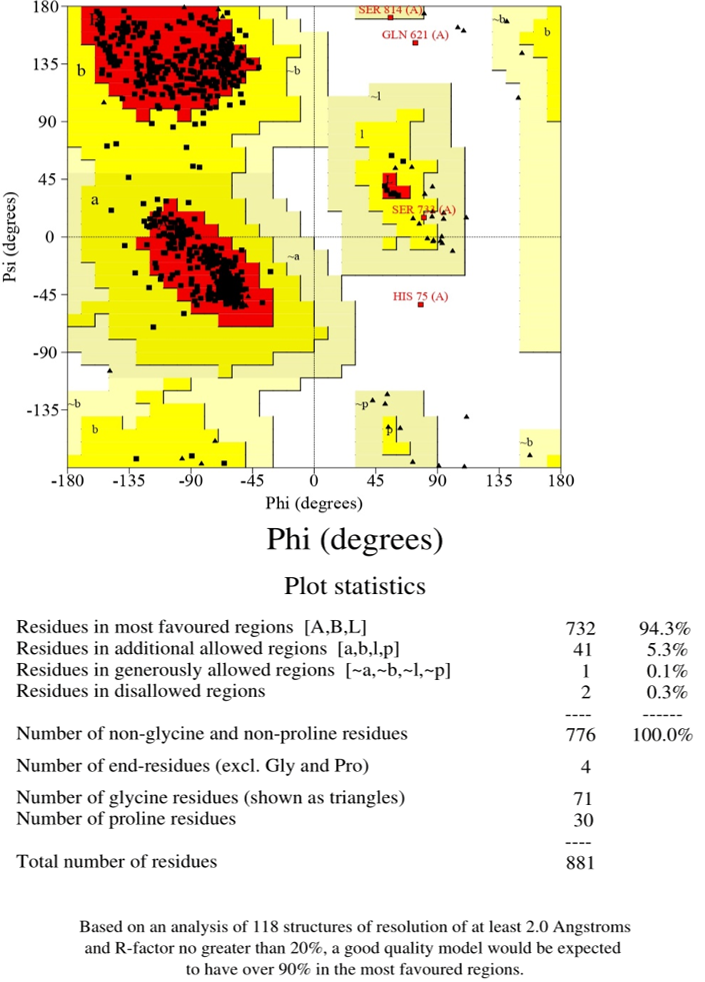

Supplement: S1 Fig — (TIF) [file pone.0301747.s001.tif]

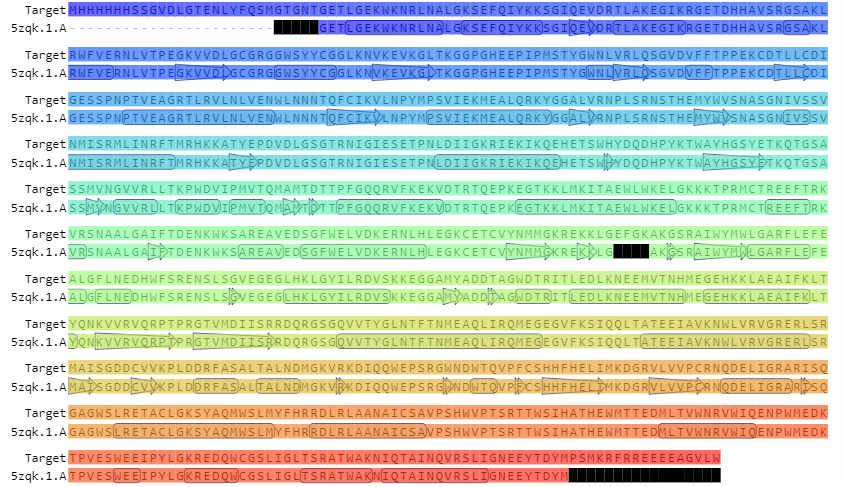

Supplement: S2 Fig — (TIF) [file pone.0301747.s002.tif]

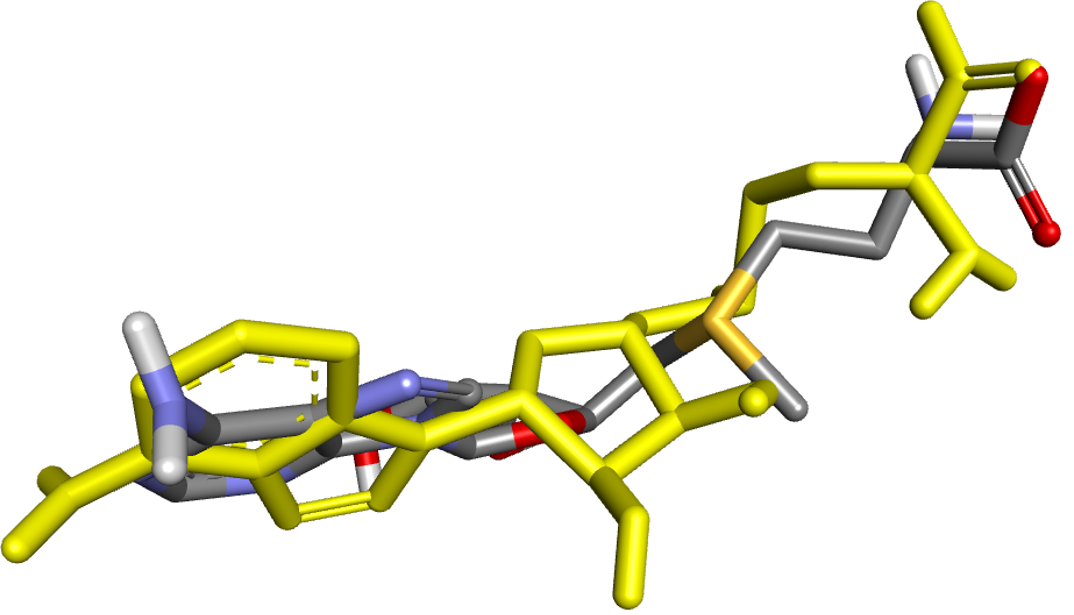

Supplement: S3 Fig — Heavy-atom RMSD = 2.618 Å. (TIF) [file pone.0301747.s003.tif]

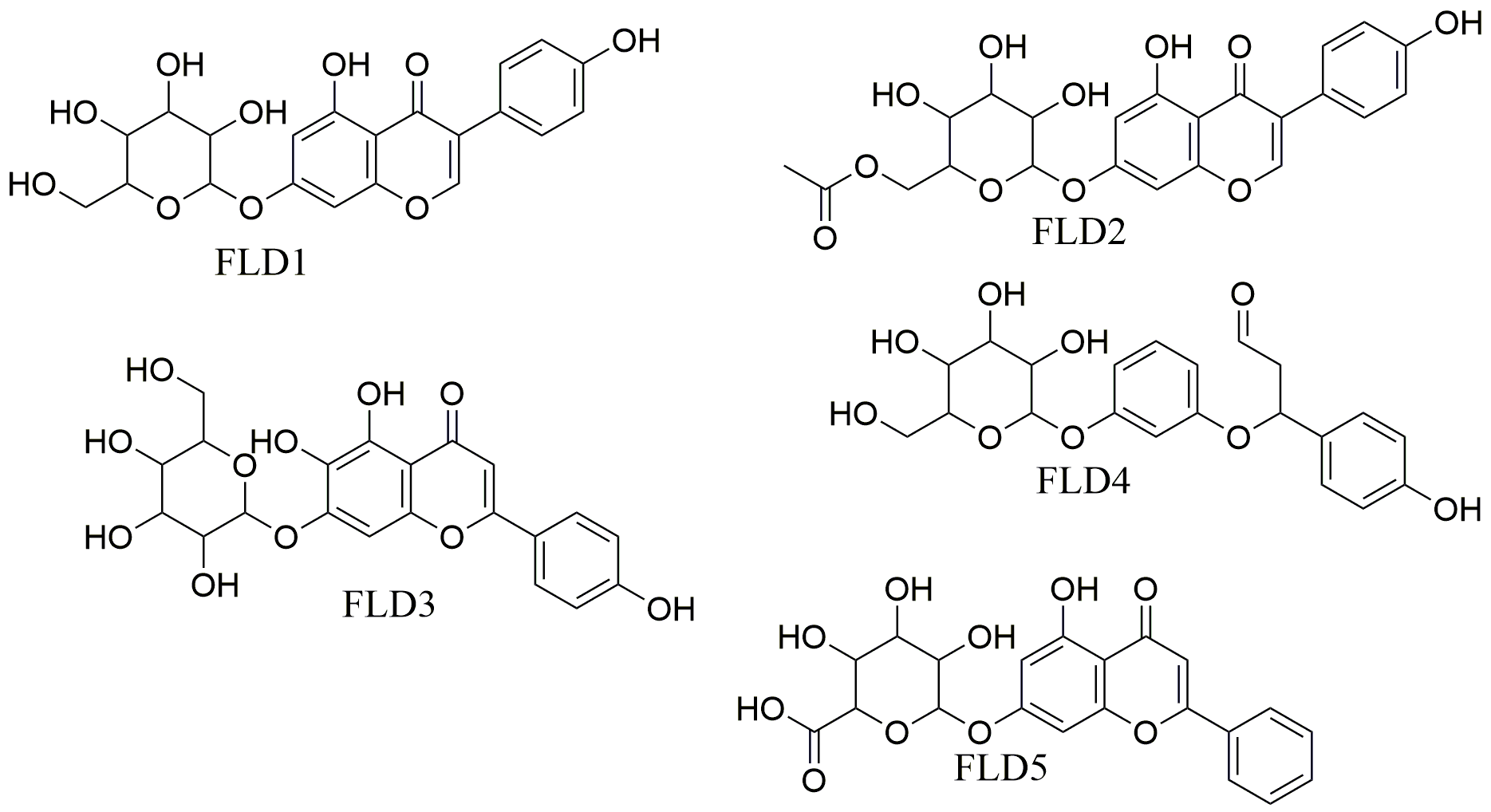

Supplement: S4 Fig — (TIF) [file pone.0301747.s004.tif]

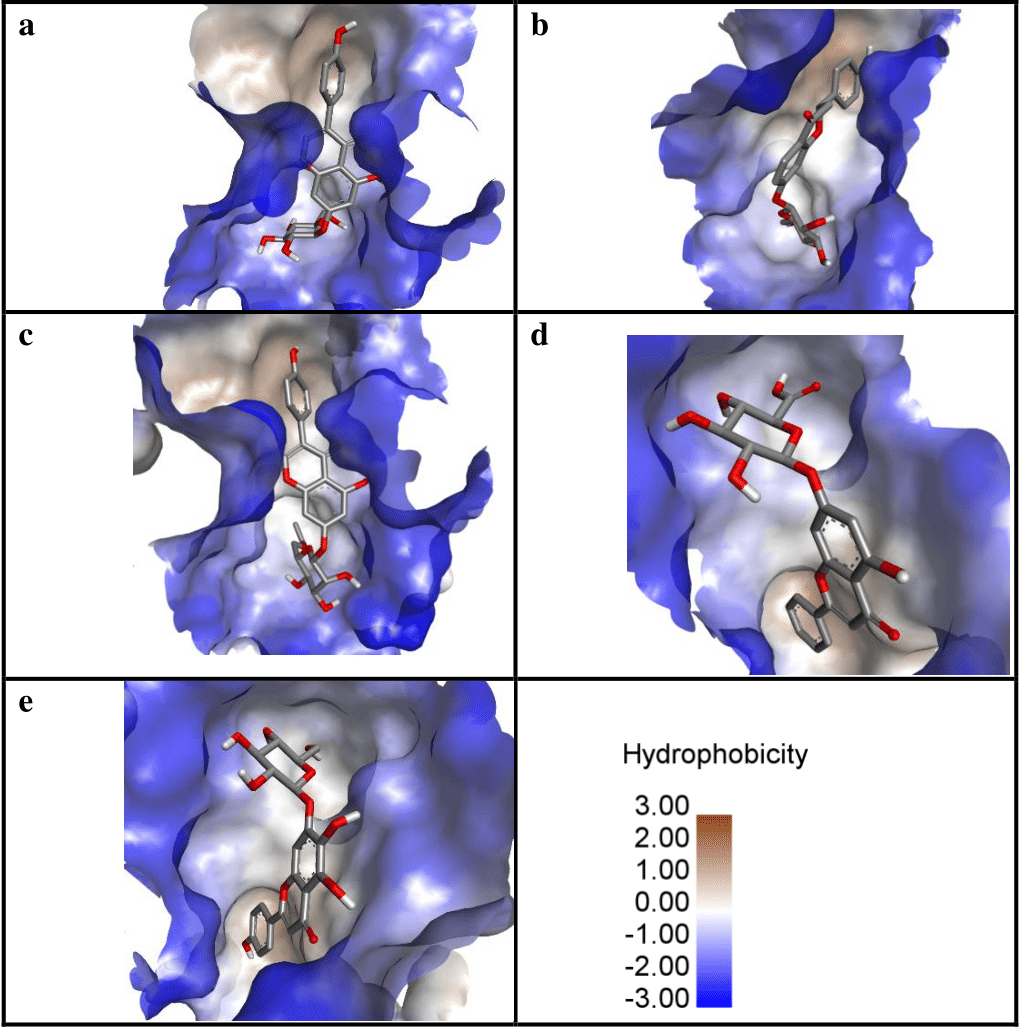

Supplement: S5 Fig — (a) FLD1 (b) FLD2 (c) FLD3 (d) FLD4, and (e) FLD5. (TIF) [file pone.0301747.s005.tif]

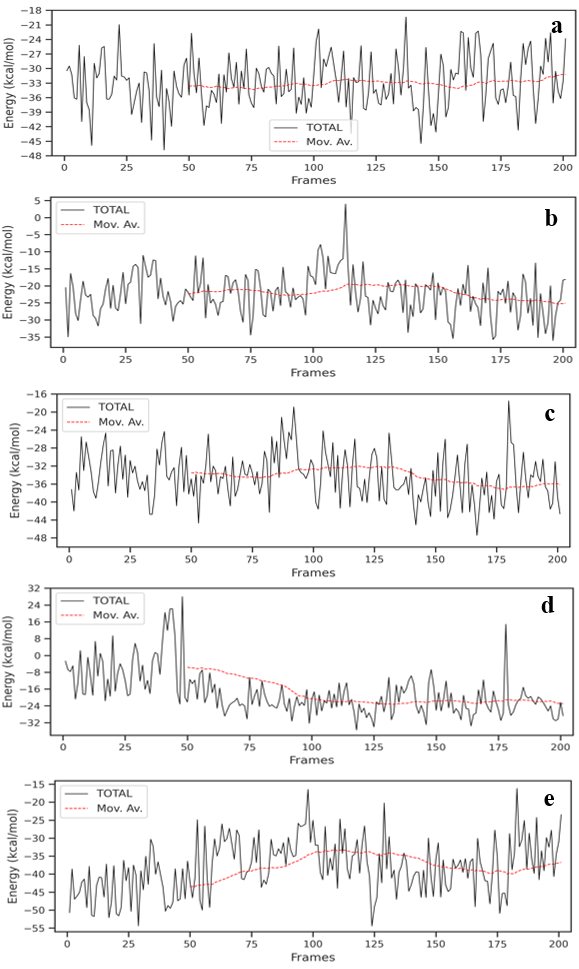

Supplement: S6 Fig — (TIF) [file pone.0301747.s006.tif]
