# Supplementary material for: In silico exploration of potent flavonoids for dengue therapeutics
Source: PLoS One. 2024 Dec 12;19(12):e0301747. doi: 10.1371/journal.pone.0301747 (PMC11637399; doi:10.1371/journal.pone.0301747)
Supplement: S1 Table — (DOCX) [file pone.0301747.s007.docx]

**S1 Table. Toxicity of the compounds from ProTox-Ⅱ.**

| Compounds | Hepatotoxicity | Carcinogenicity | Immunotoxicity | Mutagenicity | Cytotoxicity |
| --- | --- | --- | --- | --- | --- |
| FLD1 | Inactive | Inactive | Inactive | Inactive | Inactive |
| FLD2 | Inactive | Inactive | Inactive | Inactive | Inactive |
| FLD3 | Inactive | Inactive | Inactive | Inactive | Inactive |
| FLD4 | Inactive | Inactive | Inactive | Inactive | Inactive |
| FLD5 | Inactive | Inactive | Inactive | Inactive | Inactive |
| FLD6 | Inactive | Inactive | Inactive | Inactive | Inactive |
| FLD7 | Inactive | Inactive | Inactive | Inactive | Inactive |
| FLD8 | Inactive | Inactive | Inactive | Inactive | Inactive |
| FLD9 | Inactive | Inactive | Inactive | Inactive | Inactive |
| FLD10 | Inactive | Inactive | Inactive | Inactive | Inactive |
| FLD11 | Inactive | Inactive | Inactive | Inactive | Inactive |
| FLD12 | Inactive | Inactive | Inactive | Inactive | Inactive |
| FLD13 | inactive | inactive | Inactive | Inactive | Inactive |
| FLD14 | inactive | Inactive | Inactive | Inactive | Inactive |
| FLD15 | inactive | inactive | inactive | inactive | inactive |
| FLD16 | inactive | inactive | inactive | inactive | inactive |
| FLD17 | inactive | inactive | inactive | inactive | inactive |
| FLD18 | inactive | inactive | inactive | inactive | inactive |
| FLD19 | inactive | inactive | inactive | inactive | inactive |
| FLD20 | inactive | inactive | inactive | inactive | inactive |
| FLD21 | inactive | inactive | inactive | inactive | inactive |
| FLD22 | inactive | inactive | inactive | inactive | inactive |
| FLD23 | inactive | inactive | inactive | inactive | inactive |
| FLD24 | inactive | inactive | inactive | inactive | inactive |
| FLD25 | inactive | inactive | inactive | inactive | inactive |
| FLD26 | inactive | inactive | inactive | inactive | inactive |
| FLD27 | inactive | inactive | inactive | inactive | inactive |
| FLD28 | inactive | inactive | inactive | inactive | inactive |
| FLD29 | inactive | inactive | inactive | inactive | inactive |
| FLD30 | inactive | inactive | inactive | inactive | inactive |
| FLD31 | inactive | inactive | inactive | inactive | inactive |
| FLD32 | inactive | inactive | inactive | inactive | inactive |
| FLD33 | inactive | inactive | inactive | inactive | inactive |
| FLD34 | inactive | inactive | inactive | inactive | inactive |
| Reference ligand | inactive | inactive | inactive | inactive | inactive |
| Reference drug | active | inactive | inactive | inactive | inactive |
