# Supplementary material for: In silico exploration of potent flavonoids for dengue therapeutics
Source: PLoS One. 2024 Dec 12;19(12):e0301747. doi: 10.1371/journal.pone.0301747 (PMC11637399; doi:10.1371/journal.pone.0301747)
Supplement: S2 Table — (DOCX) [file pone.0301747.s008.docx]

**S2 Table. ADMET properties from ADMETlab 2.0.**

| Compounds | LogS | HIA | BBB | PBB | CYP1A2-inh | CYP1A2-sub | CYP2C19-inh | CYP2C19-sub |
| --- | --- | --- | --- | --- | --- | --- | --- | --- |
| FLD1 | -4.248 | 0.993 | 0.004 | 81.84% | 0.007 | 0.014 | 0.009 | 0.139 |
| FLD2 | -4.453 | 0.898 | 0.005 | 86.35% | 0.045 | 0.05 | 0.022 | 0.081 |
| FLD3 | -4.674 | 0.774 | 0.005 | 85.09% | 0.042 | 0.035 | 0.017 | 0.063 |
| FLD4 | -4.406 | 0.739 | 0.005 | 87.73% | 0.038 | 0.059 | 0.034 | 0.101 |
| FLD5 | -4.307 | 0.932 | 0.001 | 87.93% | 0.047 | 0.041 | 0.016 | 0.066 |
| FLD6 | -4.413 | 0.837 | 0.003 | 88.90% | 0.09 | 0.083 | 0.031 | 0.353 |
| FLD7 | -4.23 | 0.692 | 0.003 | 89.11% | 0.043 | 0.071 | 0.026 | 0.241 |
| FLD8 | -4.343 | 0.901 | 0.002 | 88.57% | 0.047 | 0.046 | 0.019 | 0.148 |
| FLD9 | -4.549 | 0.122 | 0 | 90.70% | 0.02 | 0.079 | 0.037 | 0.767 |
| FLD10 | -4.36 | 0.235 | 0.006 | 86.40% | 0.029 | 0.055 | 0.015 | 0.089 |
| FLD11 | -4.37 | 0.613 | 0.293 | 86.03% | 0.008 | 0.041 | 0.034 | 0.666 |
| FLD12 | -4.323 | 0.892 | 0.1 | 85.37% | 0.009 | 0.036 | 0.026 | 0.682 |
| FLD13 | -4.438 | 0.197 | 0.148 | 76.00% | 0.06 | 0.044 | 0.084 | 0.154 |
| FLD14 | -4.241 | 0.884 | 0.031 | 91.34% | 0.01 | 0.037 | 0.029 | 0.604 |
| FLD15 | -4.714 | 0.564 | 0.017 | 85.90% | 0.037 | 0.065 | 0.045 | 0.294 |
| FLD16 | -4.583 | 0.795 | 0.007 | 84.16% | 0.031 | 0.056 | 0.013 | 0.141 |
| FLD17 | -4.491 | 0.824 | 0.01 | 85.27% | 0.03 | 0.087 | 0.021 | 0.476 |
| FLD18 | -5.544 | 0.21 | 0 | 89.44% | 0.156 | 0.391 | 0.105 | 0.17 |
| FLD19 | -4.76 | 0.846 | 0.019 | 84.00% | 0.044 | 0.057 | 0.025 | 0.29 |
| FLD20 | -3.53 | 0.845 | 0.017 | 91.10% | 0.155 | 0.041 | 0.023 | 0.057 |
| FLD21 | -3.78 | 0.841 | 0.017 | 89.60% | 0.211 | 0.039 | 0.023 | 0.053 |
| FLD22 | -4.832 | 0.681 | 0.051 | 82.94% | 0.029 | 0.094 | 0.025 | 0.656 |
| FLD23 | -4.438 | 0.197 | 0.148 | 76.00% | 0.06 | 0.044 | 0.084 | 0.154 |
| FLD24 | -3.059 | 0.012 | 0 | 97.40% | 0.019 | 0.073 | 0.058 | 0.814 |
| FLD25 | -4.028 | 0.907 | 0.021 | 87.11% | 0.01 | 0.033 | 0.023 | 0.531 |
| FLD26 | -4.26 | 0.558 | 0.005 | 87.49% | 0.037 | 0.087 | 0.023 | 0.532 |
| FLD27 | -3.892 | 0.976 | 0.005 | 94.47% | 0.024 | 0.03 | 0.023 | 0.095 |
| FLD28 | -4.001 | 0.959 | 0.039 | 92.31% | 0.021 | 0.032 | 0.026 | 0.27 |
| FLD29 | -4.339 | 0.896 | 0.001 | 87.68% | 0.055 | 0.045 | 0.018 | 0.063 |
| FLD30 | -4.137 | 0.972 | 0.007 | 95.64% | 0.026 | 0.033 | 0.03 | 0.222 |
| FLD31 | -3.987 | 0.955 | 0.011 | 94.72% | 0.022 | 0.034 | 0.029 | 0.23 |
| FLD32 | -3.919 | 0.8 | 0.059 | 94.52% | 0.034 | 0.036 | 0.041 | 0.317 |
| FLD33 | -3.89 | 0.482 | 0.016 | 90.88% | 0.578 | 0.05 | 0.034 | 0.052 |
| FLD34 | -3.262 | 0.859 | 0.041 | 88.84% | 0.011 | 0.043 | 0.013 | 0.361 |
| native ligand | -3.339 | 0.101 | 0.557 | 51.49% | 0.007 | 0.059 | 0.029 | 0.064 |
| Reference drug | -6.007 | 0.004 | 0.097 | 99.72% | 0.443 | 0.881 | 0.099 | 0.863 |
