# Supplementary material for: In silico exploration of potent flavonoids for dengue therapeutics
Source: PLoS One. 2024 Dec 12;19(12):e0301747. doi: 10.1371/journal.pone.0301747 (PMC11637399; doi:10.1371/journal.pone.0301747)
Supplement: S3 Table — (DOCX) [file pone.0301747.s009.docx]

**S3 Table. Toxicity from ADMElab 2.0.**

| Compounds | hERG | ROA | SkinSen | EC | EI | Respiratory |
| --- | --- | --- | --- | --- | --- | --- |
| FLD1 | 0.081 | 0.385 | 0.187 | 0.003 | 0.013 | 0.817 |
| FLD2 | 0.032 | 0.18 | 0.167 | 0.003 | 0.027 | 0.34 |
| FLD3 | 0.042 | 0.113 | 0.219 | 0.003 | 0.022 | 0.368 |
| FLD4 | 0.008 | 0.333 | 0.232 | 0.003 | 0.034 | 0.411 |
| FLD5 | 0.214 | 0.056 | 0.253 | 0.003 | 0.074 | 0.211 |
| FLD6 | 0.017 | 0.067 | 0.087 | 0.003 | 0.072 | 0.34 |
| FLD7 | 0.036 | 0.078 | 0.223 | 0.003 | 0.021 | 0.127 |
| FLD8 | 0.072 | 0.101 | 0.179 | 0.003 | 0.037 | 0.468 |
| FLD9 | 0.04 | 0.207 | 0.88 | 0.003 | 0.092 | 0.102 |
| FLD10 | 0.014 | 0.383 | 0.229 | 0.003 | 0.015 | 0.156 |
| FLD11 | 0.027 | 0.516 | 0.094 | 0.003 | 0.02 | 0.92 |
| FLD12 | 0.03 | 0.265 | 0.092 | 0.003 | 0.027 | 0.874 |
| FLD13 | 0.061 | 0.26 | 0.269 | 0.003 | 0.105 | 0.105 |
| FLD14 | 0.02 | 0.402 | 0.153 | 0.003 | 0.034 | 0.916 |
| FLD15 | 0.009 | 0.723 | 0.181 | 0.003 | 0.028 | 0.544 |
| FLD16 | 0.013 | 0.216 | 0.103 | 0.003 | 0.015 | 0.235 |
| FLD17 | 0.011 | 0.796 | 0.147 | 0.003 | 0.012 | 0.447 |
| FLD18 | 0.017 | 0.213 | 0.396 | 0.003 | 0.202 | 0.075 |
| FLD19 | 0.019 | 0.504 | 0.117 | 0.003 | 0.026 | 0.459 |
| FLD20 | 0.195 | 0.039 | 0.782 | 0.003 | 0.196 | 0.063 |
| FLD21 | 0.11 | 0.076 | 0.67 | 0.003 | 0.134 | 0.085 |
| FLD22 | 0.015 | 0.924 | 0.08 | 0.003 | 0.011 | 0.542 |
| FLD23 | 0.061 | 0.26 | 0.269 | 0.003 | 0.105 | 0.105 |
| FLD24 | 0.001 | 0.216 | 0.558 | 0.003 | 0.056 | 0.967 |
| FLD25 | 0.027 | 0.214 | 0.248 | 0.003 | 0.044 | 0.801 |
| FLD26 | 0.013 | 0.815 | 0.17 | 0.003 | 0.012 | 0.489 |
| FLD27 | 0.007 | 0.142 | 0.286 | 0.003 | 0.037 | 0.934 |
| FLD28 | 0.01 | 0.19 | 0.151 | 0.003 | 0.023 | 0.941 |
| FLD29 | 0.086 | 0.1 | 0.197 | 0.003 | 0.045 | 0.324 |
| FLD30 | 0.005 | 0.312 | 0.276 | 0.003 | 0.028 | 0.951 |
| FLD31 | 0.006 | 0.292 | 0.268 | 0.003 | 0.027 | 0.953 |
| FLD32 | 0.016 | 0.238 | 0.132 | 0.003 | 0.043 | 0.869 |
| FLD33 | 0.038 | 0.18 | 0.864 | 0.003 | 0.428 | 0.039 |
| FLD34 | 0.005 | 0.135 | 0.254 | 0.003 | 0.023 | 0.858 |
| Reference ligand | 0.003 | 0.506 | 0.82 | 0.003 | 0.018 | 0.926 |
| Reference drug | 0.004 | 0.46 | 0.822 | 0.009 | 0.189 | 0.964 |
