# Supplementary material for: In silico exploration of potent flavonoids for dengue therapeutics
Source: PLoS One. 2024 Dec 12;19(12):e0301747. doi: 10.1371/journal.pone.0301747 (PMC11637399; doi:10.1371/journal.pone.0301747)
Supplement: S4 Table — (DOCX) [file pone.0301747.s010.docx]

**S4 Table. ADMET properties from pkCSM server.**

| Compounds | P-glycoprotein sustrate | P-glycoprotein I Inhibitor | P-glycoprotein II inhibitor | Renal OCT2 substrate |
| --- | --- | --- | --- | --- |
| FLD1 | Yes | No | No | No |
| FLD2 | Yes | No | No | No |
| FLD3 | Yes | No | No | No |
| FLD4 | Yes | No | No | No |
| FLD5 | Yes | No | No | No |
| FLD6 | Yes | No | No | No |
| FLD7 | Yes | Yes | Yes | No |
| FLD8 | Yes | No | No | No |
| FLD9 | Yes | No | No | No |
| FLD10 | Yes | No | No | No |
| FLD11 | Yes | No | No | No |
| FLD12 | Yes | No | Yes | No |
| FLD13 | Yes | No | No | No |
| FLD14 | Yes | No | No | No |
| FLD15 | Yes | Yes | Yes | No |
| FLD16 | Yes | No | No | No |
| FLD17 | Yes | No | No | No |
| FLD18 | Yes | No | No | No |
| FLD19 | Yes | No | No | No |
| FLD20 | Yes | No | No | No |
| FLD21 | Yes | No | No | No |
| FLD22 | Yes | Yes | No | No |
| FLD23 | Yes | No | No | No |
| FLD24 | Yes | No | No | No |
| FLD25 | Yes | Yes | No | No |
| FLD26 | Yes | Yes | Yes | No |
| FLD27 | Yes | No | No | No |
| FLD28 | Yes | yes | No | No |
| FLD29 | Yes | Yes | Yes | No |
| FLD30 | Yes | No | No | No |
| FLD31 | Yes | No | No | No |
| FLD32 | Yes | No | No | No |
| FLD33 | Yes | No | No | No |
| FLD34 | Yes | No | No | No |
| Native ligand | Yes | No | No | No |
| Reference drug | Yes | No | No | No |
