# Supplementary material for: In silico exploration of potent flavonoids for dengue therapeutics
Source: PLoS One. 2024 Dec 12;19(12):e0301747. doi: 10.1371/journal.pone.0301747 (PMC11637399; doi:10.1371/journal.pone.0301747)
Supplement: S5 Table — (DOCX) [file pone.0301747.s011.docx]

**S5 Table. Binding affinity of compounds from different servers.**

| **S.N.** | **Flavonoids**  **(PubChem CID)** | **Code** | **Binding Affinity (kcal/mol) from DockThor** | **Binding Affinity (kcal/mol) from SwissDock** |
| --- | --- | --- | --- | --- |
| 1 | 5284639 | FLD1 | −8.962 | −8.659 |
| 2 | 22288010 | FLD2 | −8.412 | −8.034 |
| 3 | 14332446 | FLD3 | −8.118 | −8.118 |
| 4 | 13473703 | FLD4 | −8.063 | −8.053 |
| 5 | 14135334 | FLD5 | −8.053 | −8.421 |
| 6 | 5213 | FLD6 | −7.774 | −7.597 |
| 7 | 13873666 | FLD7 | −7.739 | −7.700 |
| 8 | 137796321 | FLD8 | −7.658 | −7.320 |
| 9 | 51666248 | FLD9 | −7.661 | −7.432 |
| 10 | 74819354 | FLD10 | −7.658 | −7.811 |
| 11 | 53398699 | FLD11 | −7.625 | −7.641 |
| 12 | 72747690 | FLD12 | −7.604 | −7.793 |
| 13 | 12004622 | FLD13 | −7.579 | −7.432 |
| 14 | 623002 | FLD14 | −7.576 | −6.697 |
| 15 | 73192461 | FLD15 | −7.199 | −6.730 |
| 16 | 5358913 | FLD16 | −7.449 | −6.769 |
| 17 | 5489114 | FLD17 | −7.443 | −7.523 |
| 18 | 4183640 | FLD18 | −7.369 | −6.125 |
| 19 | 25202038 | FLD19 | −7.301 | −6.350 |
| 20 | 51136398 | FLD20 | −7.260 | −6.012 |
| 21 | 3321055 | FLD21 | −7.199 | −6.017 |
| 22 | 3733033 | FLD22 | −7.199 | −7.720 |
| 23 | 5317025 | FLD23 | −7.176 | −6.869 |
| 24 | 73829903 | FLD24 | −7.199 | −6.720 |
| 25 | 3483754 | FLD25 | −7.176 | −6.123 |
| 26 | 74977902 | FLD26 | −7.069 | −6.978 |
| 27 | 4789 | FLD27 | −7.059 | −6.−872 |
| 28 | 42607667 | FLD28 | −7.045 | −7.568 |
| 29 | 6169038 | FLD29 | −6.971 | −7.162 |
| 30 | 131752198 | FLD30 | −6.884 | 7.532 |
| 31 | 5080434 | FLD31 | −7.257 | −6.021 |
| 32 | 20979874 | FLD32 | −7.041 | −6.035 |
| 33 | 102421333 | FLD33 | −6793 | −6.023 |
| 34 | 73009474 | FLD34 | −6.646 | −6.123 |
| 35 | 440426607 | Native ligand (SAM) | −5.887 | −6.212 |
| 36 | 5018304 | Diclifenac sodium | −6.885 | −6.554 |
