# Supplementary material for: In silico exploration of potent flavonoids for dengue therapeutics
Source: PLoS One. 2024 Dec 12;19(12):e0301747. doi: 10.1371/journal.pone.0301747 (PMC11637399; doi:10.1371/journal.pone.0301747)
Supplement: S6 Table — Orientation and location of ligands relative to protein backbone are to be monitored; ligand is shown in ball and stick model and protein in cartoon representation. (DOCX) [file pone.0301747.s012.docx]

**S6 Table. Snapshots of five protein-ligand complexes at different times during MDS.** Orientation and location of ligands relative to protein backbone are to be monitored; ligand is shown in ball and stick model and protein in cartoon representation.

**0 ns 50 ns 100 ns 150 ns 200 ns**

| 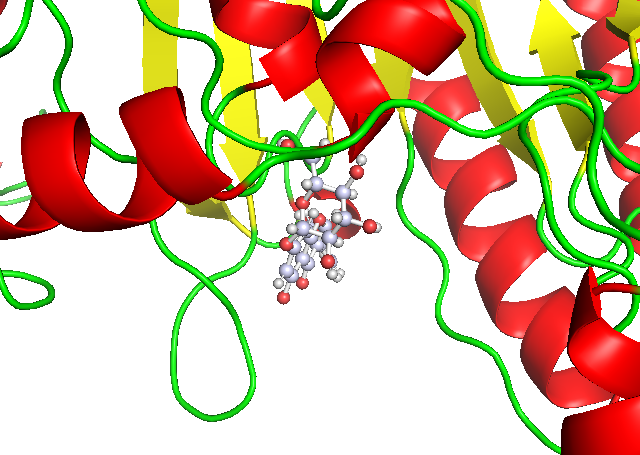**complex1** | 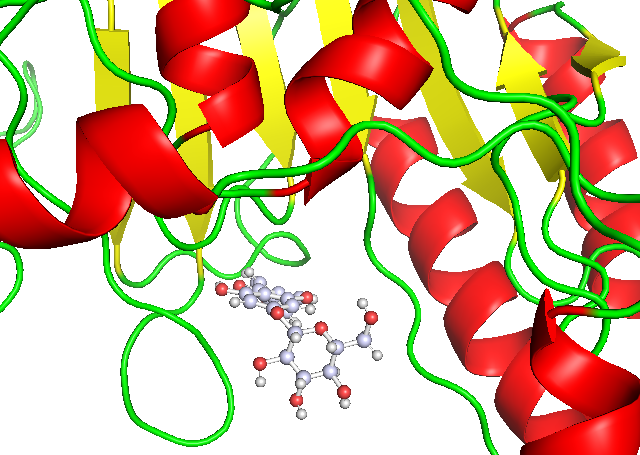 | 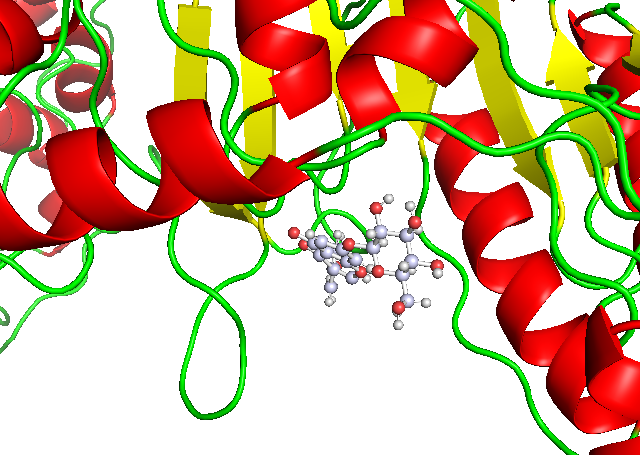 | 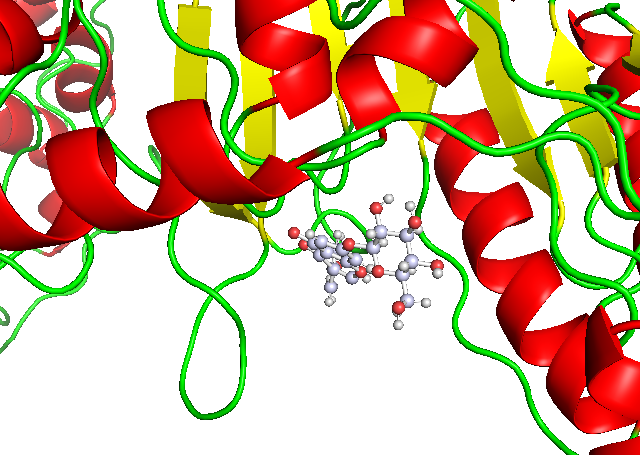 | 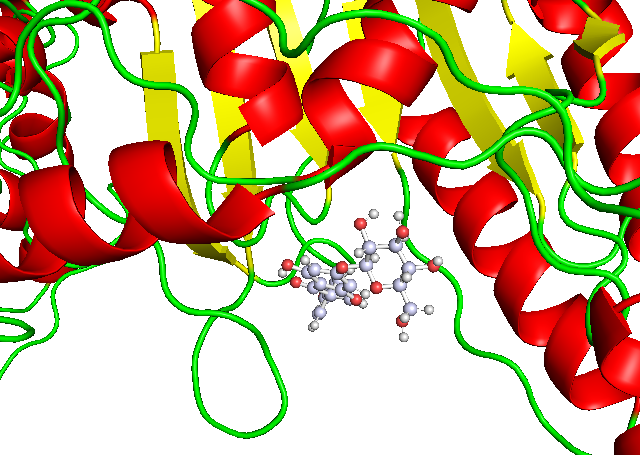 |
| --- | --- | --- | --- | --- |
| 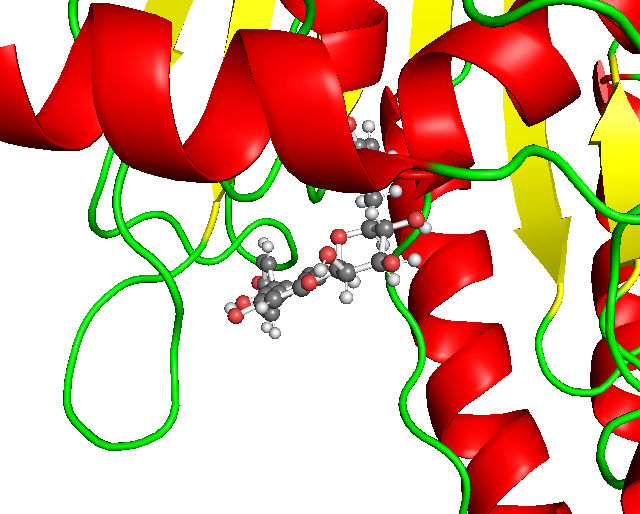**complex2** | 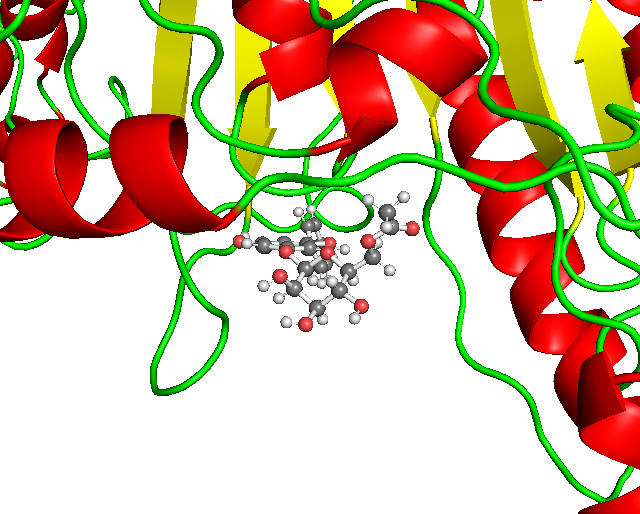 | 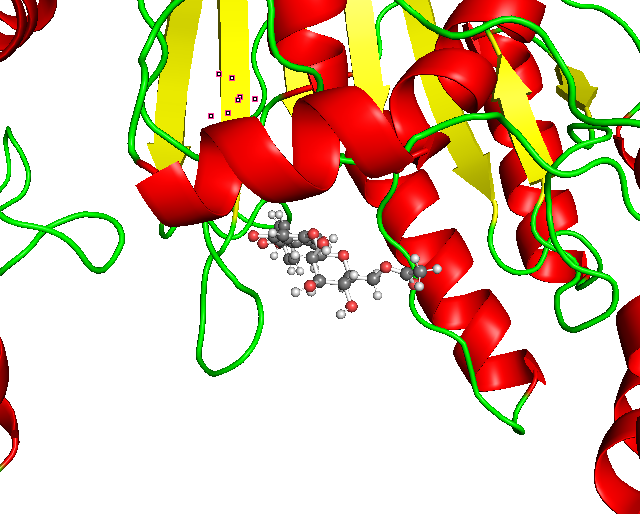 | 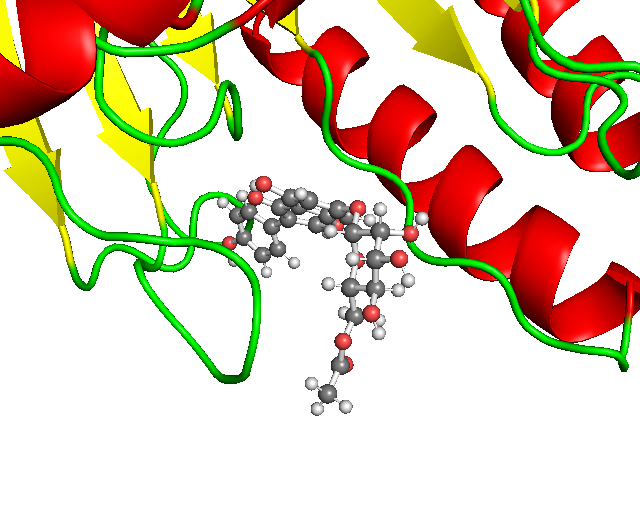 | 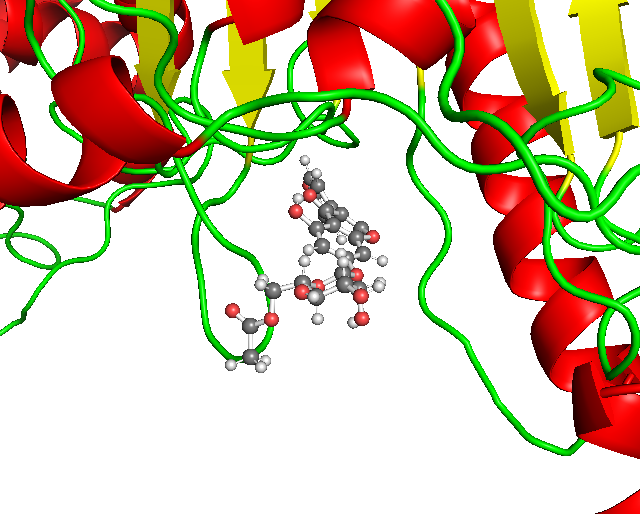 |
| 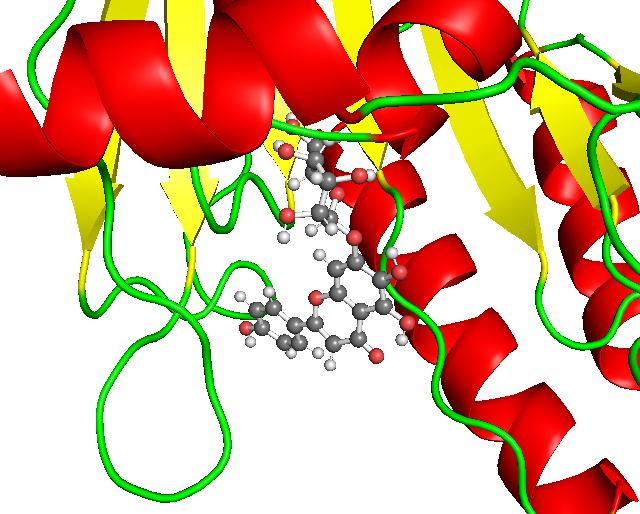**complex3** | 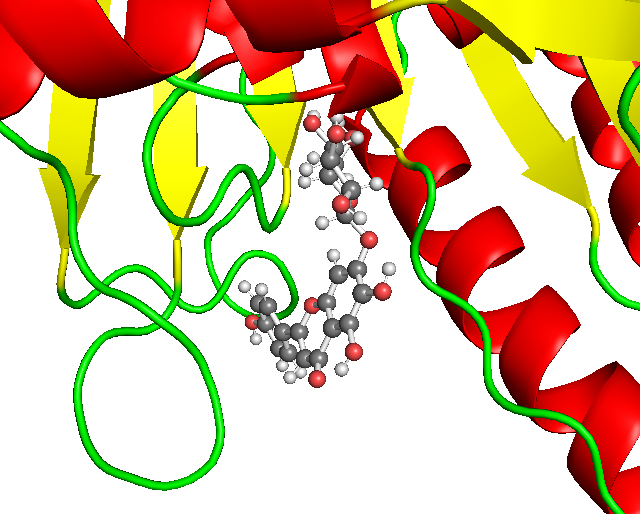 | 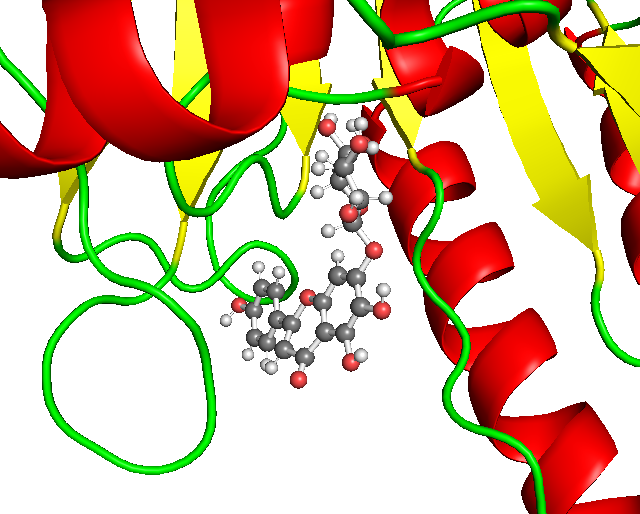 | 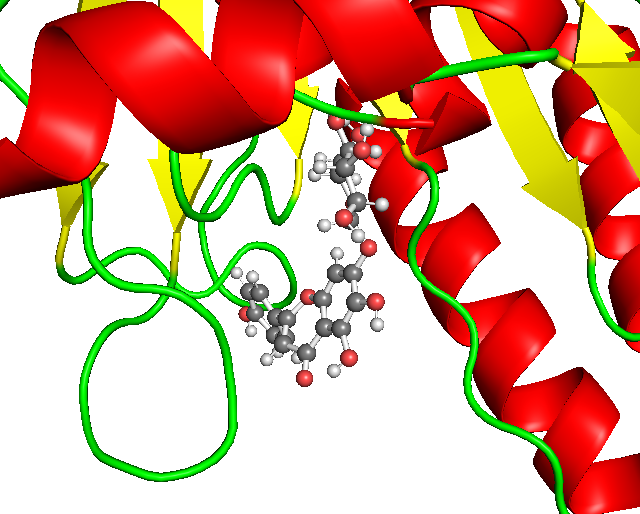 | 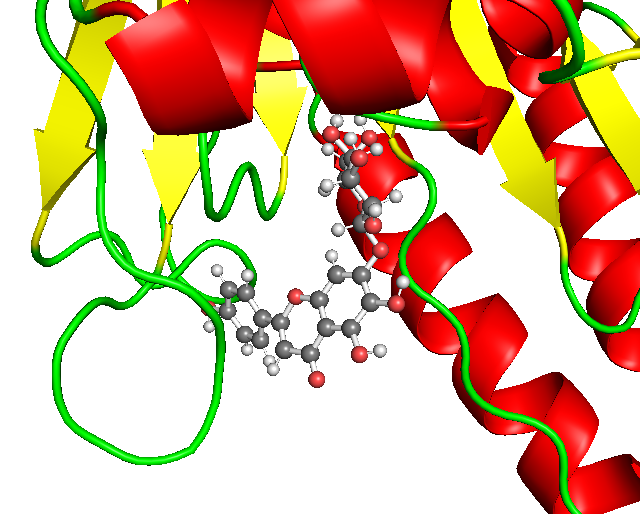 |
| 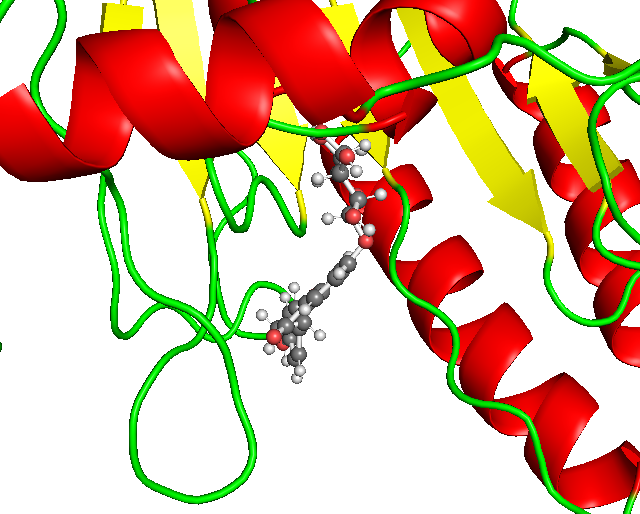**complex4** | 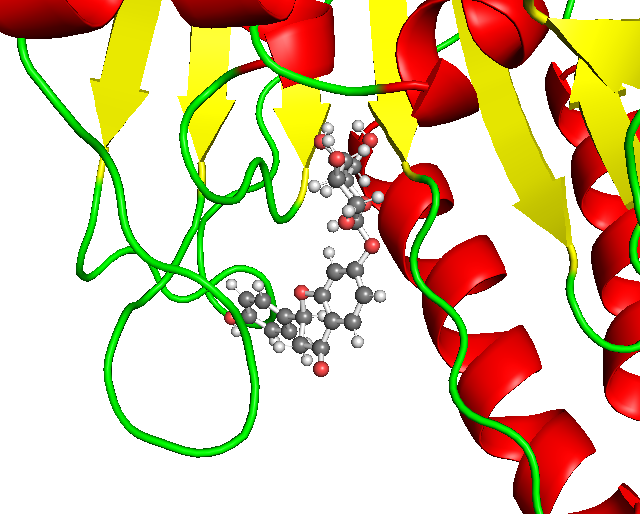 | 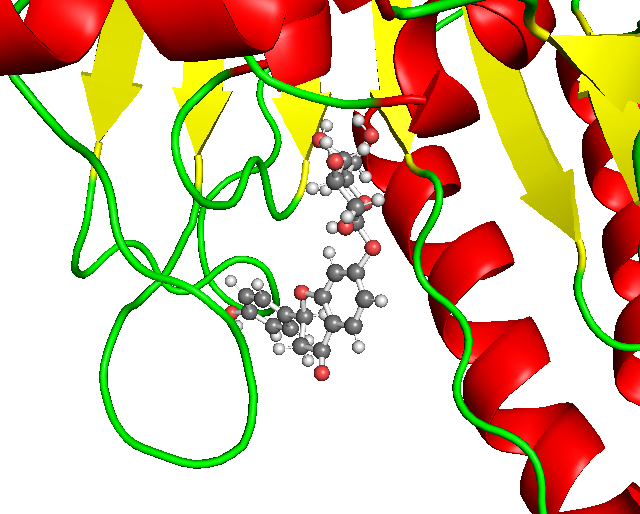 | 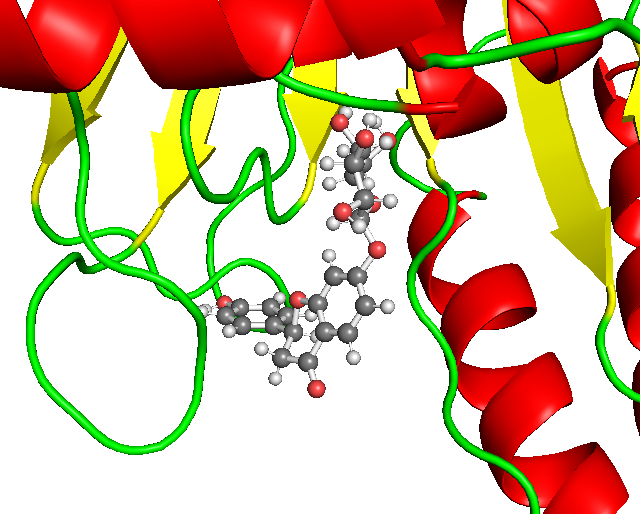 | 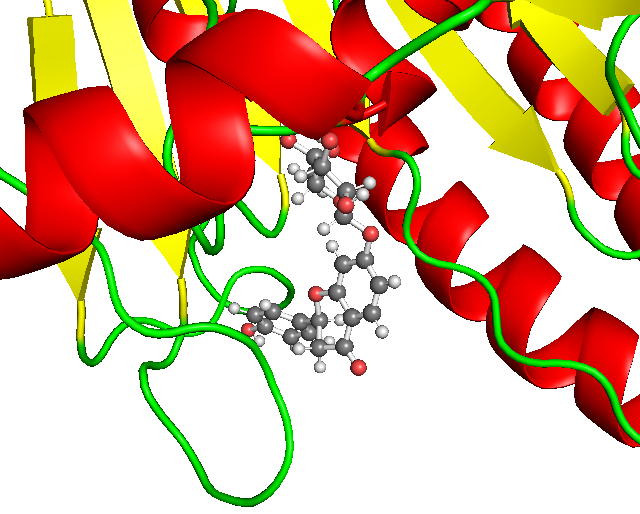 |
| 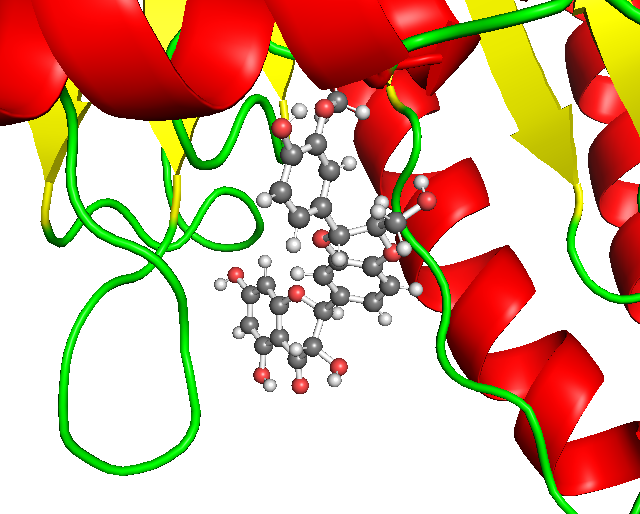**complex5** | 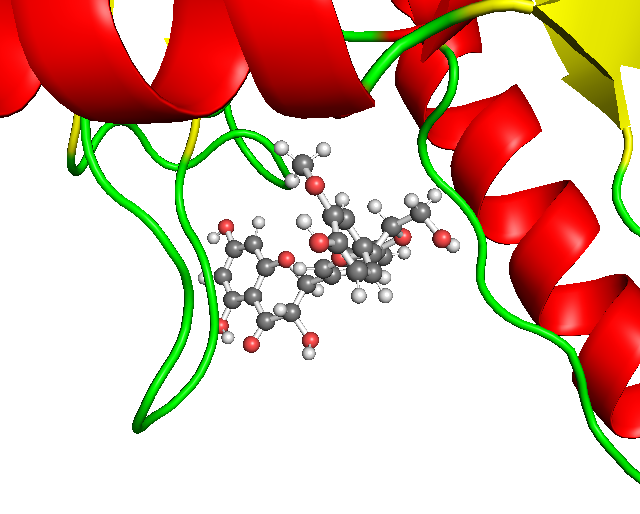 | 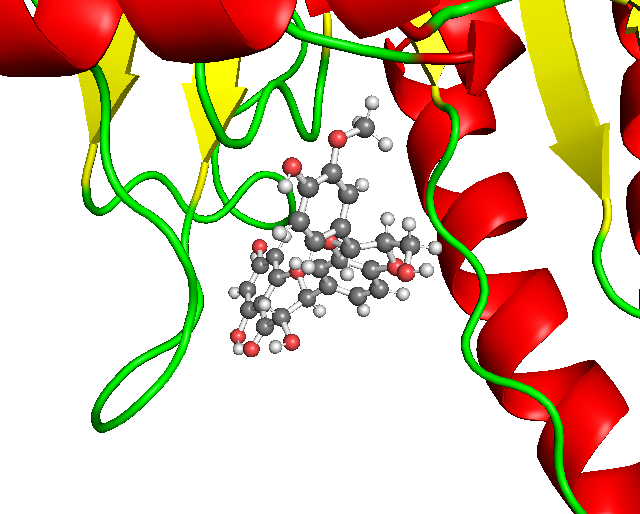 | 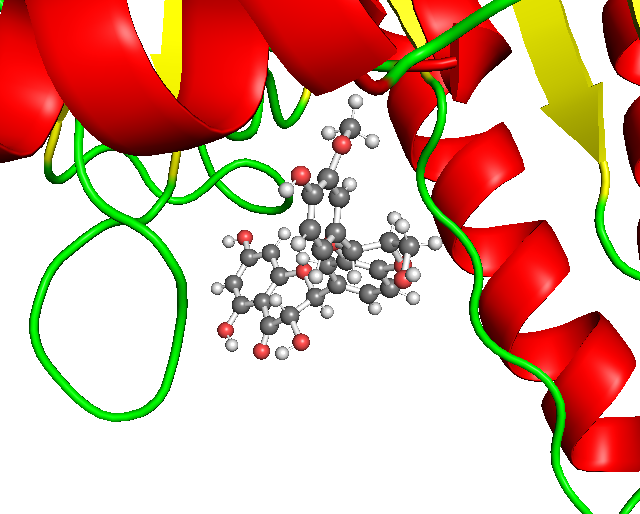 | 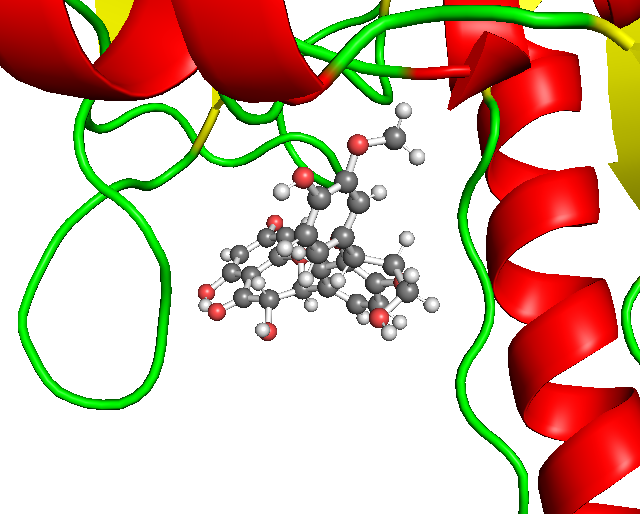 |
