# Supplementary material for: In silico exploration of potent flavonoids for dengue therapeutics
Source: PLoS One. 2024 Dec 12;19(12):e0301747. doi: 10.1371/journal.pone.0301747 (PMC11637399; doi:10.1371/journal.pone.0301747)
Supplement: Attachment — Submitted filename: Response to Reviewers.docx [file pone.0301747.s013.docx]

13/09/2024

To,

The Academic Editor

**PLOS ONE**

Subject: Responses to the comments and suggestions

Dear Editor,

Herein, we are submitting our revised research manuscript entitled **"*In silico* exploration of potent flavonoids for dengue therapeutics"** for publication in your esteemed journal, **PLOS ONE.** We have addressed the reviewer’s and your comments with track changes to the best of our knowledge and effort. We want to express our sincere gratitude for the insightful suggestions and for providing ample time for a thorough revision.

**Journal requirements:**

**When submitting your revision, we need you to address these additional requirements.**

1. **Please ensure that your manuscript meets PLOS ONE's style requirements, including those for file naming.**

**Response:** The manuscript is presented in the format specified by the journal with proper naming of associated files.

2. **We suggest you thoroughly copyedit your manuscript for language usage, spelling, and grammar. If you do not know anyone who can help you do this, you may wish to consider employing a professional scientific editing service.**

**Response:** We have thoroughly corrected the manuscript in terms of grammar and language.

3. **Please include captions for your Supporting Information files at the end of your manuscript, and update any in-text citations to match accordingly. Please see our Supporting Information guidelines for more information:**

**Response:** We have included captions of all the Supporting Information files at the end of the manuscript.

**Editor’s Comments:**

We appreciate the editor's insightful remarks. Your concern has been addressed in the following ways:
 **I have received the reviewer comments on the manuscript entitled“ In silico exploration of potent flavonoids for dengue therapeutics” submitted by Phunyal and coworkers. The manuscript discusses possible flavonoids for the inhibition of the dengue protein. My suggestions are listed below.**

**1. Add a detailed discussion on the background of dengue cases. Please refer very recent articles:** [**https://doi.org/10.1002/hsr2.1831**](https://doi.org/10.1002/hsr2.1831)**;** [**https://doi.org/10.1002/hsr2.2089**](https://doi.org/10.1002/hsr2.2089)

**Response:** We have included the suggested articles in the manuscript (Ref. no. 2 and 5).

**2. Make a discussion on why the study on arboviral diseases is necessary in recent days.**

**Response:** The relevant discussion has been added in the introduction section (first paragraph).

**3. Very old references are used. Total 78 references. Make upto 50 references. It is a research article.**

**Response:** Thank you for your suggestion. Now, we have reduced to 59 references. Multiple attempts to decrease this number resulted in the omission of relevant text and scientific content of the research.

**4. Please see in the introduction---"Currently, there is a lack of specific antiviral medications for treating dengue infection. SanofiPasteur’s tetravalent dengue vaccine (CYD-TDV, Dengvaxia) has been approved for clinical use in several countries [8,9]. It has raised concerns regarding its overall efficacy in the general population [10,11]. Therefore proper, effective, and safe therapeutics for the treatment and cure of dengue are still lacking."----all the references are old. Here written currently???? Refer recent data and reference**

**Response:** We have updated the manuscript with recent articles to address the outdated data (Ref. no. 9 and 10 are the newer ones.)

**5. As per your findings, which one/two is best, discuss with data in conclusion.**

**Response:** Our computational work showed FLD4 ligand as the best molecule and has been mentioned accordingly in the conclusion. Relevant interpretation has been done in R&D section.

**6. Due to lack of specific antiviral medications for treating dengue infection, please discuss other control measures in the conclusion (2/3 lines).**

**Response:** We have clearly addressed the concern in the conclusion section of the manuscript.

**7. Make the references as per journal style.**

**Response:** The manuscript references have been formatted according to the journal’s style to the best of our effort. Some inconsistencies have been rectified manually.

**Reviewers' comments:

Reviewer's Responses to Questions**

**Comments to the Author**

**Review Comments to the Author

Please use the space provided to explain your answers to the questions above. You may also include additional comments for the author, including concerns about dual publication, research ethics, or publication ethics. (Please upload your review as an attachment if it exceeds 20,000 characters)**

**Response:** Done.

Reviewer 1:

We appreciate reviewer 1’s insightful remarks. Your concern has been addressed thoroughly.

1. **A decrease in platelet counts below 100000 cells/micro L, and a decrease in WBC and Neutrophil are diagnostic parameters of dengue fever (Potts JA, Rothman AL. Clinical and laboratory features that distinguish dengue from other febrile illnesses in endemic populations. Trop Med Int Health. 2008 Nov;13(11):1328-40. doi: 10.1111/j.1365-3156.2008.02151.x. Epub 2008 Sep 16. PMID: 18803612; PMCID: PMC2756447.).
   It should be included in the Introduction.**

**Response:** The relevant text has been added in the introduction section (Ref. no. 7).

2. **The Authors may also refer to Mohapatra et al (2023) while discussing the role of flavonoids as antiviral agents along with ref.24. (Mohapatra, P. K., Chopdar, K. S., Dash, G. C., Mohanty, A. K., & Raval, M. K. (2023). In silico screening and covalent binding of phytochemicals of Ocimum sanctum against SARS-CoV-2 (COVID 19) main protease. Journal of Biomolecular Structure and Dynamics, 41(2), 435–444.** [**https://doi.org/10.1080/07391102.2021.2007170**](https://doi.org/10.1080/07391102.2021.2007170)**)
   Authors may also consider referring the following article along with Ref25
   Thames et al. Synthesis and biological evaluation of novel flexible nucleoside analogues
   that inhibit flavivirus replication in vitro. Bioorganic & Medicinal Chemistry 28 (2020) 115713**

**Response:** We have incorporated all the recommended articles (Ref. no. 18 and 19) in the manuscript.

3. **Discussion of Sinefungin a natural mimic of SAM/SAH is relevant.
   Ref Yebra et al. The effect of sinefungin and synthetic analogues on RNA and DNA methyltransferases from Streptomyces. The Journal of Antibiotics 1991; 44 (10): 1141- 1147.**

**Response:** The chemical structures of sinefungin, dehydrosinefungin, SAM, and SAH are similar. Sinefungin was not included in our study. Since only the cognate ligand, SAM occupied the orthosteric pocket of the receptor with a PDB ID of 5ZQK, other species were not considered. The competitive inhibitors to SAM were explored in this research.

**4. Respiratory toxicity of FLD1 (0.817) needs discussion on whether FLD1 to be included in short listed prodrugs.**

**Response:** The prediction shows that it is toxic in nature and is to be verified by multiple experiments. Relevant text has been added in the ADMET subsection and is applicable to any molecule under any toxicity endpoints. This generalization encompasses all the molecules studied and all the parameters predicted.

**5. The resolution of Figures appearing in the MS is not acceptable. It needs improvement.**

**Response:** Now, we have improved the resolution and clarity of the figures according to **PLOS One** guidelines to the best of our skills.

Reviewer 2:

We appreciate reviewer 2’s valuable comments. Your concern has been addressed in the following ways:

**The MS entitled“ In silico exploration of potent flavonoids for dengue therapeutics” by Phunyal et al discusses possible flavonoids for the inhibition of the dengue protein. The MS is well composed and written.
 Few general comments need to be addressed by the authors.**

**1. "The database of ca. 2000 molecules was screened based on toxicity criteria from the ADMETlab2.0 server "– Why only "2000 "phyto compounds are only screened?**

**Response:** A database of 3D similar structures of a skeleton structure (scaffold) of flavonoid was generated. It comprised of nearly 2000 different chemical species and were used for the computational calculations in our work. Among different classes of organic compounds, flavonoids are bioactive in nature and tend to possess effective therapeutic values. Ninfali et al. have reported the possibilities of flavonoids against dengue and zika viruses. This has formed the basis of our studies and it has helped to create a database comprising of significant number of relevant molecules suitable for the available computational facilities. Future work with larger datasets has been planned.

**2. Why homology modelling of the receptor is required? The blast result may be provided in the supplementary section In material method section. “homology modeling” the template name is missing.**

**Response:** The answer to this question has been included in subsection 2.2. The BLAST result is attached in a supplementary file. The template name has been incorporated.

**3. To find the potential candidates for dengue therapeutics molecular docking was performed using Autodock Vina software (version 1.1) was used for molecular docking [49]
Sentenced need to be reconstructed**

**Response:** Appropriate changes have been made.

**4. Grid size in the Molecular Dynamics section in material methods should be mentioned.**

Response: The required information has been added in subsection 2.5.

**5. Figures should have better clarity and Fig 09 required be re-adapted with better clarity and the Y- axis in specific.**

**Response:** High-resolution figures have been used. The Y-axis for the top and bottom figures corresponds to ligand RMSD and backbone RMSD, respectively, and has been mentioned in the caption.

**6. Authors should look into the spelling and grammatical mistakes.**

**Response:** The whole manuscript has been thoroughly revised in terms of spelling errors, grammatical inconsistencies, sentence construction, clarity of information, and flow of proper scientific contents.

Reviewer 3:

We appreciate reviewer 3’s concerns. It has been addressed in the following ways:

**1. Figures seem very low resolution without proper description of legends. Four- five figures will be standard.**

**Response:** High-resolution figures have been uploaded. The legends have been clarified. The number of figures has been reduced without compromising the scientific content of the article

**2. Please apply three to fo[u]r docking server and validate results to get best output.**

**Response:** Molecular docking from three different algorithms has been performed and validation has been done accordingly. It has been added in the supporting information.

**3. Please clear about control group run. It is mandatory for docking and simulation.**

**Response:** The molecular docking of the control ligand has been performed. However, due to the lack of force field (CHARMM, OPLS, GROMOS etc.) of it, molecular dynamics simulation could not be performed. The literature lacks this calculation and could not be cited or compared. An acceptable RMSD (not good though) for SAM was obtained and formed the basis for molecular docking calculations.

**4. 500ns MD simulation is highly appreciated, if it is not possible please add more data analysis based on statistic.**

**Response:** Thank you for your suggestion. Due to the unavailability of computational resources, a 500 ns MD simulation could not be performed. However, RPDF data has been extracted from the current trajectories and has been incorporated as subsection 3.5.7. This has supported the findings and could probably provide an alternate solution for the suggestion.

**5. Introduction
Please add few lines for epidemiology:**[**https://doi.org/10.1002/hsr2.1831**](https://doi.org/10.1002/hsr2.1831)**;**

**Response:** We have addressed this comment in the manuscript (ref. no 2).

**6. Please add references: The single stranded positive-sense RNA genome is its genetic material, approximately 11 kb in size. DOI: 10.1097/MS9.0000000000000623**

**Response:** We have included this article (Ref. no. 12) in the manuscript. **7. Please add research gaps regarding this research.**

**Response:** Now, we have added some points showing the current research gap in dengue therapeutics in the introduction section.

**8. Method:
For protein structure validation, the SAVES v6.0 web server (**[**https://saves.mbi.ucla.edu/**](https://saves.mbi.ucla.edu/)**) with three modules namely, ERRAT [37], VERIFY3D [38], and PROCHECK [39] (Ramachandran plot) were used to identify protein sequences and assessed the quality of protein structure. Please add accuracy level in the result part.**

**Response:** This comment has been clarified in 3.2. subsection.

**9. Please perform two or more docking servers.**

**Response:** Molecular docking from three different algorithms has been performed and validation has been done. Same as comment 2 above.

**10. Result**

**Please add data in supplementary files.**

**Response:** We have performed additional molecular docking using two docking servers and have added results to the supplementary files (S5 Table).

**10. Please follow same format for number and digit writing: The overall quality factor from the ERRAT module of the protein was found to be 94.2197%, and from the VERIFY module, the structure of the protein was verified (87.02%)
Response:** We have addressed this comment in the manuscript, ensuring consistent formatting for numbers and digits.

**11. Figures
Increase resolution. Suggestion: Add ppt slides or follow journal rules. Please lessen figures and transfer in supply.**

**Response:** We have followed the journal guidelines, increased the image resolution, and added the figures accordingly to the best of our effort.

Once again, we appreciate the feedback that has helped to refine the manuscript and uplift it to an acceptable quality concerning the scientific content along with the mode of presentation. We would be happy to revise it further and our apologies in case of any oblivious errors.

Thank you once again.

Sincerely,

Authors
