# Supplementary material for: In silico exploration of potent flavonoids for dengue therapeutics
Source: PLoS One. 2024 Dec 12;19(12):e0301747. doi: 10.1371/journal.pone.0301747 (PMC11637399; doi:10.1371/journal.pone.0301747)
Supplement: Attachment — Submitted filename: Response to Reviewers.docx [file pone.0301747.s014.docx]

October 08, 2024

To,

The Academic Editor

**PLOS ONE**

Subject: Responses to the comments and suggestions (second revision)

Dear Editor,

Herein, we are submitting the revised research manuscript entitled **"*In silico* exploration of potent flavonoids for dengue therapeutics".** We have addressed all the reviewer comments with track changes to the best of our knowledge and effort. Our sincere gratitude to the reviewers for the insightful suggestions that has helped to refine and rectify the manuscript.

**Journal requirements:**

Please review your reference list to ensure that it is complete and correct. If you have cited papers that have been retracted, please include the rationale for doing so in the manuscript text, or remove these references and replace them with relevant current references. Any changes to the reference list should be mentioned in the rebuttal letter that accompanies your revised manuscript. If you need to cite a retracted article, indicate the article’s retracted status in the References list and also include a citation and full reference for the retraction notice.

**Response:** We have reviewed the reference list for ensuring that it is complete and correct.

**Review Comments to the Author**

Please use the space provided to explain your answers to the questions above. You may also include additional comments for the author, including concerns about dual publication, research ethics, or publication ethics. (Please upload your review as an attachment if it exceeds 20,000 characters)

**Reviewer #1: The revision has improved the quality of the article. Authors have addressed all the queries satisfactorily.**

**Response:** Thank you for your positive feedback.

**Reviewer #2: The authors addressed the required queries raised in the 1st round review process. The MS may be accepted in the present form.**

**Response:** We appreciate your recommendation for accepting the manuscript in its current form.

**Reviewer #3: 
Please decrease the number of figures:
1. Amalgamate fig a1 and 2**

**Response:** We have moved Fig 2 to the supplementary file as S3 Fig to decrease the figure count. We could not amalgamate Fig 1 (part of the Introduction) and Fig 2 (part of the Results and Discussion) because these are present at different manuscript sections.

**2. Delete fig 3 or move in supplementary file**

**Response:** Fig 3 has been moved to the supplementary file.

**3. Move fig 5 to supplementary file**

**Response:** Fig 5 has been transferred to the supplementary file.

**4. Fig 6-10 (add all of them like 5a, 5b, 5c, and so on)**

**Response:** Thank you for your suggestion. Figures 6 to 9 (Fig 10 already has a number of figures) have been consolidated and presented as a single figure with subparts (according to journal style). Consequently, the reduction in the number of figures in the main manuscript has been done.

Once again, we appreciate the feedback that has helped to enhance the scientific content along with the mode of presentation.

Sincerely,

Jhashanath Adhikari Subin and the team
